# Transient up-regulation of miR-155-3p by lipopolysaccharide in primary human monocyte-derived macrophages results in RISC incorporation but does not alter TNF expression

**DOI:** 10.12688/wellcomeopenres.15065.2

**Published:** 2019-10-03

**Authors:** Rachel E. Simmonds

**Affiliations:** 1 Department of Microbial Sciences, University of Surrey, Guildford, GU2 7XH, UK; 2 Cytokine and Signal Transduction Laboratory, Kennedy Institute of Rheumatology, London, W6 8LH, UK

**Keywords:** miRNAs, miR-155, Toll-like receptor signalling, primary human cells, monocyte-derived macrophages, TNF, mycolactone

## Abstract

**Background:** The innate immune response is a tightly regulated process that reacts rapidly in response to pathogen-associated molecular patterns (PAMPs) such as lipopolysaccharide (LPS). Evidence is accumulating that microRNAs contribute to this, although few studies have examined the early events that constitute the “primary” response.

**Methods:** LPS-dependent changes to miRNA expression were studied in primary human monocyte-derived macrophages (1°MDMs). An unbiased screen by microarray was validated by qPCR and a method for the absolute quantitation of miRNAs was also developed, utilising 5’ phosphorylated RNA oligonucleotide templates. RNA immunoprecipitation was performed to explore incorporation of miRNAs into the RNA-induced silencing complex (RISC). The effect of miRNA functional inhibition on TNF expression (mRNA and secretion) was investigated.

**Results:** Of the 197 miRNAs expressed in 1°MDMs, only five were induced >1.5-fold. The most strongly induced was miR-155-3p, the partner strand to miR-155-5p, which are both derived from the MIR155HG/BIC gene (pri-miR-155). The abundance of miR-155-3p was induced transiently ~250-fold at 2-4hrs and then returned towards baseline, mirroring pri-miR-155. Other PAMPs, IL-1β, and TNF caused similar responses. IL-10, NF-κB, and JNK inhibition reduced these responses, unlike cytokine-suppressing mycolactone. Absolute quantitation revealed that miRNA abundance varies widely from donor-to-donor, and showed that miR-155-3p abundance is substantially less than miR-155-5p in unstimulated cells. However, at its peak there were 446-1,113 copies/cell, and miR-155-3p was incorporated into the RISC with an efficiency similar to miR-16-5p and miR-155-5p. Inhibition of neither miRNA affected TNF secretion after 2hrs in 1°MDMs, but technical challenges here are noted.

**Conclusions:** Dynamic regulation of miRNAs during the primary response is rare, with the exception of miR-155-3p. Further work is required to establish whether its low abundance, even at the transient peak, is sufficient for biological activity and to determine whether there are specific mechanisms determining its biogenesis from miR-155 precursors

## Introduction

In recent years, microRNAs have emerged as important post-transcriptional regulators of gene expression ^1^. They are mostly produced first as primary transcripts (pri-miRNAs; usually from pol II-dependent promoters) that are subsequently processed by DROSHA/DGCR8 in the microprocessor to form ~70nt hairpin structures (pre-miRNAs) that have the potential to encode two different miRNAs. The pre-miRNAs are exported from the nucleus, processed further by DICER into mature miRNAs and loaded into the RNA-induced silencing complex (RISC) to carry out their regulatory functions. The fine detail of this canonical pathway of miRNA biogenesis and other non-canonical pathways have been extensively reviewed elsewhere ^1– 3^ The two miRNAs that can be encoded on a pre-miRNA have historically been known as the ‘guide’ and ‘passenger’ strands, with the latter being referred to as the “star” miRNA. Early on, these were thought to be degraded with only the ‘guide’ strand going on to perform gene regulatory functions ^4^. An increasing body of evidence has revealed this not to be the case, and in miRbase, the public repository for miRNA sequence annotation, the two strands are now referred to as -5p and -3p, indicating whether the miRNA is located at the 5’ or 3’ end of the pre-miRNA ^5^. This recognises that each form has the potential to play a regulatory role depending on its expression in different cells, or in response to stimuli.

Inflammation is one of the most rapid and tightly regulated processes of the immune response, and includes the activation of cells by pathogen- and damage-associate molecular patterns (PAMPs and DAMPs) that are recognised by pattern recognition receptors (PRRs) including the Toll-like receptors (TLRs) ^6^. For example, lipopolysaccharide (LPS) is recognised by a complex of TLR4 with CD14 antigen and MD2/LY96 (Myeloid Differentiation Protein-2/Lymphocyte Antigen 96). Following activation, at least two well-defined signalling pathways are activated, including MyD88-IRAK-4-TRAF6-NFκB and TRIF-TRAF3-IRF3. Many cell types can respond to PAMPs and DAMPs however, inflammation in the innate immune response is primarily mediated by monocytes and macrophages of the myeloid lineage, in particular by production of the alarm cytokine TNF. It is well-established that the response can be divided into two phases. A primary response, which does not require new protein synthesis, and a secondary response that does. For instance, many of the cytokines secreted during the primary response can stimulate cytokine receptors by autocrine/paracrine mechanisms further stimulating the cells.

Several microRNAs are commonly found to be upregulated during disease states involving inflammation ^7^, and at least two of these (miR-155-5p and miR-146a-5p) are regulated by NFκB, downstream of TLR signalling. Indeed, miR-155 is one of the most intensively studied immune microRNAs ^8^. This well conserved miRNA is encoded by the MIR155HG gene, previously known as non-coding B cell integration cluster (BIC), which has three exons (in humans) the third of which contains the miR-155 pre-miRNA. Overexpression of MIR155HG causes spontaneous lymphoma in mice ^9^. On the other hand, MIR155HG knockout causes defects in germ centre formation and Ig class switching, leading to immunodeficiency ^10^. The function of miR-155 as a master switch in inflammation has been extensively reviewed elsewhere ^7, 8^. Briefly, induction of miR-155-5p by inflammatory stimuli including LPS in myeloid cells (monocytes, macrophages and dendritic cells), B and T cells has been widely reported.

The existence and function of the -3p miRNA for miR-155 has been less intensively studied and is more controversial. While miR-155-3p has been present in miRbase for many years (originally annotated as miR-155*), it was Zhou *et al*. who first described TLR7-dependent induction of both miR-155-5p and miR-155-3p in plasmacytoid dendritic cells ^11^. It has since been shown to be induced by cytokines (TNF and IL-1β/IFNγ) and TLR3 ligands in astrocytes ^12^, during LPS stimulation of trophoblasts ^13^, and during *Mycobacterium tuberculosis* infection of monocyte-derived DCs ^14^. In murine systems, miR-155-3p has been shown to be upregulated in M1 (LPS and IFNγ) bone marrow-derived macrophages ^15^, as well as in infiltrating T helper cells in experimental autoimmune encephalomyelitis ^16^. As well as immune pathways, miR-155-3p has been reported to be regulated in other physiological process, including downregulation during cardiogenesis from embryonic stem cells ^17^ and in human glioblastoma cells during hypoxia ^18^. It was also identified in a methylated form in mantle cell lymphoma (MCL; an aggressive B-cell non-Hodgkin's lymphoma), and demethylation resulted in increased expression, revealing tumour suppressing properties ^19^. Despite this, the TargetScan database ^20^ includes miR-155-3p as a “not confidently identified miRNA”. Here, an arbitrary cut-off of ~1,000 copies/cell of an miRNA is defined as that minimum required for biological function, although no mechanistic data is provided in support of this.

Most of the studies mentioned above, similar to the miRNA field more widely, utilise relative expression of miRNAs using a range of techniques such as q-RTPCR, miRNAseq and/or microarrays that result in finding of fold-changes under different conditions. While such analyses are highly informative, most do not provide information on the abundance of the miRNAs, itself known to vary widely. One way of deriving this information is to perform absolute quantitation of the miRNAs (AQ-miRNA) by PCR; however, there is a lack of specific published protocols describing the methodology. Here, a widely applicable method using 5’ phosphorylated RNA oligonucleotides as the template in two-step miRNA expression assays is described.

This manuscript reports some historical data, in which a miRNA microarray was performed to investigate changes to miRNA abundance during the primary immune response. The rationale was that since cytokines are produced by myeloid cells in an inflammatory “burst” with signalling events and key transcripts being increased transiently, any miRNA involvement in this pathway would also need to be rapid, within minutes/hours of TLR activation. Hence, the model chosen was primary human monocyte-derived macrophages (1°MDM) stimulated with LPS to specifically examine TLR signalling pathway activation rather than macrophage polarisation. This revealed the transient upregulation of miR-155-3p during the early stages of the innate immune response. Abundance of miR-155-3p was around 10-fold lower than miR-155-5p but at its peak it was shown to be incorporated into the RISC. It remains unclear whether miR-155-3p has biological function because of its low abundance and lack of functional data obtained. In contrast to previous studies ^21, 22^, here neither miR-155-5p nor miR-155-3p could be shown to influence TNF secretion by 1°MDM. This might be explained by the fact that most other studies looked at longer-term responses, rather than the primary response as was the focus here.

## Methods

### Reagents

RPMI 1640 cell culture medium and FBS were from PAA. TLR-grade LPS from *Escherichia coli* was from Sigma Aldrich. Other TLR ligands were: poly(:IC), MALP-2, Flagellin and R848 (all from Alexis Biochemicals), and Pam _3_Cys-Ser(Lys) _4_.3HCl (Pam _3_Cys) from Invivogen. Cytokines (M-CSF, GM-CSF, IL-1β, IL-10 and TNF) were from Peprotech. The inhibitors SB203580, PD98059 and SP600125 were from Calbiochem. Cycloheximide (CHX) was from Sigma Aldrich. Mycolactone A/B purified from *Mycobacterium ulcerans* was a gift from Prof Pamela Small (University of Tenessee). Synthetic mycolactone A/B was a gift from Prof Yoshito Kishi (Harvard University). All reagents were tested for contamination with endotoxin using the *limulus* amebocyte lysate assay (Lonza) and found to have <0.1 U/ml LPS.

### Primary human monocyte-derived macrophages, cell culture, RNA extraction, gene expression assays

Primary human monocytes were obtained from plateletphoresis residues purchased from the North London Blood Transfusion Service. Mononuclear cells were routinely isolated by Ficoll-Hypaque centrifugation followed by elutriation as previously described ^23, 24^. Monocyte-derived macrophages (MDMs) were obtained by differentiating the cells for 4 days with 10 ng/ml M-CSF in complete RPMI with 10% FCS but lacking antibiotics. The data in this manuscript was derived from at least 10 independent enrichments and differentiations.

1°MDMs were harvested then re-plated at 1x10 ^6^ cells/ml and were routinely stimulated with 100 ng/ml LPS. Other TLR ligands were used at different concentrations: poly(:IC); 20 µg/ml, Pam _3_Cys; 10 ng/ml, MALP-2; 30 ng/ml, Flagellin; 10 ng/ml and R848; 1 µg/ml. All cytokines were used at 10 ng/ml. When used, the final concentration of other inhibitors were SB203580 (p38; 1 µM), PD98059 (ERK, 20 µM), SP600125 (JNK, 10 µM), CHX (translation, 10 µg/ml), and PSI (proteasome inhibitor/ NFκB, 5 µM) were pre-incubated with the cells for 1 hr prior to stimulation. Mycolactone A/B was used a different concentrations depending on its provenance, and at concentrations shown to completely inhibit TNF secretion ^23, 25^; 10ng/ml for natural mycolactone A/B and 200ng/ml for synthetic mycolactone.

Samples were harvested by washing twice with sterile PBS and the addition of 600 µl miRvana Lysis/homogenisation buffer (Ambion/Life technologies). Total RNA was extracted using the miRvana miRNA purification kits (Ambion/Life Technologies) according to the manufacturer’s instructions. Quantitation and QC was by nanodrop. Samples were routinely investigated for the abundance of TNF mRNA and cytokine secretion (by in-house ELISA ^23, 24^), as an external control for expected behaviour of the cells in response to ligands and/or inhibitors. Changes in steady-state gene expression were assessed in one-step RT-PCR reactions using RNA-to-Ct reagents and Taqman gene expression probes (both Applied Biosystems) using the following probes: TNF; Hs00174128_m1, pri-miR-155; Hs01374569_m1, GAPDH, #4352934-1101034. Cycling conditions were 50°C for 15 minutes, 95°C for 2 minutes then 40 cycles of 95°C for 15 seconds and 58°C for 25 seconds on an ABI 7900HT instrument (Applied Biosystems) in either duplicate or triplicate with a 384-well block and reaction volumes of 6 µl.

### miRNA microarray

Pooled total RNA extracted from the macrophages of four independent human donors were used in Exiqon v10.0 dual-label miRcury LNA arrays, which provides 100% coverage of miRbase v10 (719 mature human miRNAs). RNA samples for the array were examined by Bioanalyser (Agilent) for quality control purposes; RIN values were routinely >9. A dual-label approach was employed, in which each test sample was labelled with Hy3, and a “common reference” sample (obtained by combining equal quantities of RNA from each of the six experimental data points) was labelled with Hy5. This approach ensured that all possible miRNAs produced at any experimental condition should be present in the common reference pool. This was considered vital in the experimental design, as many miRNAs were expected to be induced by LPS. The microarray was performed by Exiqon as a contracted service. Background subtraction used a convolution model; Normexp with offset value 1 ^26^. Normalisation was by LOWESS regression. Spots where no signal above background was detected were removed.

Gene ontology for the predicted targets of miR-155-5p and miR-155-3p was performed using predicted targets obtained from miRDB ^27^ and Panther version 14.1 over-representation test ^28^. The references was *Homo sapiens* (all genes) and Panther pathways were examined using Fisher’s exact test. All significantly over-represented pathways in the predicted target list are shown.

### Method for the absolute quantitation of miRNAs

Relative changes in miRNA abundance were quantified using Taqman miRNA assays (Applied Biosystems) using the following probes: hsa-miR-155; #2623, hsa-miR-155#; #2287, hsa-miR-16; #39. These include a miRNA reverse transcription step to create a miRNA-specific cDNA that also introduces extension sequences that can be used as the template for qPCR in the second step using the TaqMan MicroRNA Reverse Transcription Kit (Applied Biosystems). The manufacturer’s recommendations were followed, with 80 ng total RNA as the input. After reverse transcription, 50 µl nuclease water was added, diluting the reactions by 4.3-fold. Subsequent qPCR was performed using an ABI 7900HT instrument in either duplicate or triplicate with a 384-well block and reaction volumes of 10 µl. Each reaction consisted of 4.5 µl of the diluted reverse-transcription reaction, 5 µl of 2x Universal PCR Master Mix, No AmpErase UNG (Applied Biosystems), 0.35 µl of the specific 20x Taqman Assay and 0.15 µl nuclease-free water. Singleplex reactions were used because multiplex reactions were found to significantly alter the ΔCts during the highly dynamic TLR4 response (for example see raw data file 5_171, available on OSF ^29^). Negative controls included separate NTCs for reverse transcription and PCR steps. Cycling conditions were 95°C for 10 minutes then 40 cycles of 95°C for 15 seconds and 60°C for 1 minute.

All PCR-based absolute quantification (AQ) approaches involve the amplification of templates at known concentrations in order to form a standard curve based on Ct values. Hence, the AQ-miRNA approach uses synthetic miRNAs (5’ phosphorylated RNA oligonucleotides, Eurofins MWG) as the template for miRNA reverse transcription reactions (see Results). These corresponded to the miRbase sequences of hsa-miR-16-5p, hsa-miR-155-5p and has-miR-155-3p ( Table 1). The oligonucleotides were re-suspended in nuclease-free water at a concentration of 100 pmol/µl (100 pM) and stored at -80°C.

**Table 1.** Sequences of synthetic miRNAs (5’ phosphorylated RNA oligonucleotides).

miRNA	Sequence
hsa-miR-155-5p	UUAAUGCUAAUCGUGAUAGGGGU
hsa-miR-155-3p	CUCCUACUAAUUAGCAUUAACA
hsa-miR-16-5p	UAGCAGCACGUAAAUAUUGGCG

To generate the standard curve, serial dilutions of the synthetic miRNAs were reverse transcribed using the corresponding RT Primer and an input range 0.001–100 fmol/reaction (in 5 µl, giving a total volume of 15 µl). This was achieved by diluting each synthetic miRNA to 20 fmol/µl (0.2 µM) then performing eight 1 in 10 dilutions in nuclease-free water. Due to the extremely low concentration of miRNAs resulting, the dilutions were made from stocks fresh each occasion. No other alterations were made to the manufacturer’s protocol and test samples were reverse transcribed in parallel with the standard curve. These were either 80 ng total RNA, or 5 µl of the total RNA obtained during RIP (equivalent to 6.25% of the total, see below). Subsequently all standards and test samples were handled in an identical manner, and absolute quantitation was performed on the ABI 7900HT with the same reaction set up as described above.

This analysis gave results with the units “fmol”. For analysis of cellular abundance of a particular miRNA, this equated to fmol/80 ng total RNA. In order to convert this value into copies/cell, the following calculation was performed:

$$\frac{copies}{cell}=fmol\;in\;qPCR\times \frac{total\;average\;yield\;RNA\;ng}{80ng}\times 6.02x10^{8}÷no.of\;cells$$

### RNA immunoprecipitation (RIP) of miRNAs loaded in the RISC

In order to determine whether miRNAs were incorporated into the RISC complex following their expression, an RNA immunoprecipitation (RIP) strategy was employed, building on the method of Wang ^30^. Here, approximately 10x10 ^6^ MDMs were treated, then washed twice on ice with ice-cold PBS then lysed with Ago lysis buffer (ALB) for 10 mins on ice, then centrifuged for 10 mins at 4°C. ALB contained 20 mM Tris (pH 7.5) 200 mM NaCl, 2.5 mM MgCl _2_, 0.5% Triton X-100, 1x protease inhibitor cocktail (Sigma) and 100 U/ml RNasin (Promega) made up in RNase free water.

The supernatant-containing the cytoplasmic fraction was pre-cleared with protein G sepharose beads [pre-treated with 0.5 mg/ml tRNA (Invitrogen) and 1 mg/ml BSA (New England Biolabs) to block non-specific binding sites] for >30 mins. Beads were removed by centrifugation and the supernatant was processed for RIP.

For RIP, precoated Protein G sepharose beads were prepared by incubation with either 2 µl of ascities fluid containing antibodies to human Argonaute proteins (2A8, kind gift of Zissimos Mourelatos, University of Pennsylvania) ^31^ or normal mouse serum (as a control, containing non-relevant IgGs), then blocked with 0.5 mg/ml tRNA and 1 mg/ml BSA, as above. Precoated beads were then incubated with the precleared supernatants for at least 1.5 hours. Beads were then washed twice with ice cold ALB, then three times with high salt/detergent ALB (ALB containing 900 mM NaCl and 1% Triton-X-100), then twice with ALB and finally once with a low-detergent ALB (ALB containing 0.005% Triton-X-100). Finally, the beads were resuspended in 250 µl 1x DNase buffer (Promega) with 200 U/ml RNasin and 0.04 U/ml DNase I and incubated at 37°C for 20 mins. After removal of the supernatant, the beads were either boiled in 1x Gel Sample Buffer for SDS-PAGE and immunoblotting according to standard techniques ^25^, or they were resuspended in 600 µl miRvana Lysis/homogenisation buffer for RNA extraction. For immunoblotting, the primary antibody was 2A8 (diluted 1 in 500), and the secondary was an HRP-conjugate polyclonal goat anti-mouse Ig (RRID AB_2617137) at 1 in 2000.

For miRNA AQ, the formula used to convert fmol to copies/per cell was:

$$\frac{\text{copies}}{\text{cell}}=\text{fmol}\;\text{in}\;\text{qPCR}\times \frac{80\text{μl}}{5\text{μl}}\times 6.02\times 10^{8}÷\text{no}.\text{of}\;\text{cells}$$

Alternatively, incorporation of miRNAs is estimated as % of input RNA, which eliminated the need for AQ miRNA. Here, 20% of the cell lysates were processed directly for total RNA before proceeding with RIP. Here, the calculation used was:

$$\;\%\text{input}=2^{-[{\text{IP}}_{\text{Ct}}-{\text{Input}}_{\text{Ct}(\text{mean})}]}\times \frac{100}{20}$$

### Inhibition of miRNAs

Both miR-155-5p and miR-155-3p were inhibited using miRCURY LNA knockdown probes (Exiqon; miR-155-5p, #410078-00; miR-155-3p, #410079-00; negative control Scramble-miR, #199002-00). Oligonucleotides were transfected into macrophages using Dharmafect 1 (DF1, Dharmacon) using a well-optimised method ^32^. Briefly, complexes of oligonucleotides in a 1/50 dilution of DF1 in OmtiMEM (Life Technologies) were prepared. Transfections took place in serum-free RPMI with no phenol red to which complexes were added in a final proportion of 75:25%. The recovery time between transfection and stimulation was 3.5 hrs. Pilot experiments showed that allowing cells to recover overnight (18hrs) resulted in loss of miRNA inhibition. After recovery, media was replaced with complete RPMI, then stimulated with LPS for 2 hrs.

### Statistical analysis

Error calculations for technical replicates of qPCR were performed using Microsoft Excel, where the errors is calculated from the combined standard deviation of the dCt, using the formula SQRT(SUMSQ[SD of housekeeping Cts]+SUMSQ[SD of Gene of Interest Cts]). The minus error adds this value to the ddCt, calculates 2 ^x^, then calculates the difference from the final relative expression. This approach takes into account the non-linear nature of qPCR data. Excel spreadsheets showing this calculation are available on OSF ^29^.

**Figure 1.**
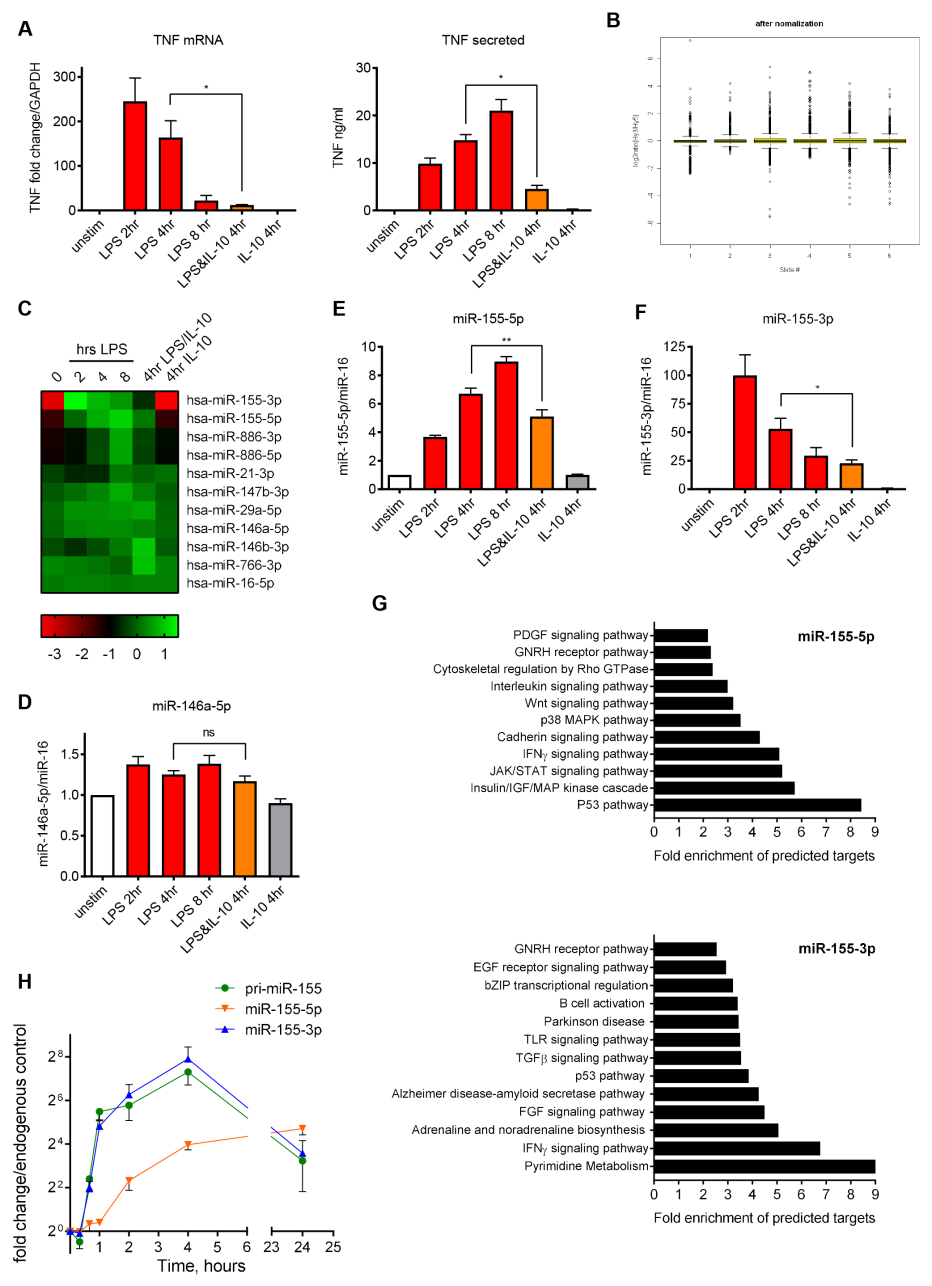
miR-155-5p and miR-155-3p are differentially regulated in primary human monocyte derived macrophages by LPS. Human 1°MDMs were exposed to 100 ng/ml LPS and/or 10 ng/ml IL-10 for different time periods, total RNA was extracted and cell culture supernatants were collected. ( **A**) Control data, showing that the cells had the expected kinetics of TNF mRNA induction and secretion in response to LPS and/or IL-10. N=4. Mean±SEM; paired t-test, *p<0.05. ( **B**) Box plot showing the array data after normalisation. ( **C**) Heat map of the miRvana microarray v.10 showing relative abundance of miRNAs compared to the common reference sample. Shown are the top 6 miRNAs up-regulated by LPS at each timepoint as well as miR-146b-3p and miR-766-3p that were up-regulated by IL-10 at 4 hours, and miR-16-5p which did not change over the timecourse. ( **D**, **E**, **F**) Validation of miR-146a-5p, miR-155-5p and miR-155-3p by relative expression with Taqman miRNA assays using RNA from 4 independent human donors. The comparator was miR-16-5p. Mean±SEM; paired t-test, *p<0.05, **P<0.01, ns P>0.05 ( **G**) Panther pathways statistically over-represented within the predicted targets of miR-155-5p (701 genes) and miR-155-3p (423 genes) in miRDB. The fold enrichment over the *Homo sapiens* reference dataset is displayed. ( **H**) Relative changes to the expression of pri-miR-155 (MIR155HG), miR-155-5p, and miR-155-3p and over time; the endogenous control for the miRNAs was miR-16-5p, for pri-miR-155 it was GAPDH. Mean±SEM of n=5–9 independent human donors.

**Figure 2.**
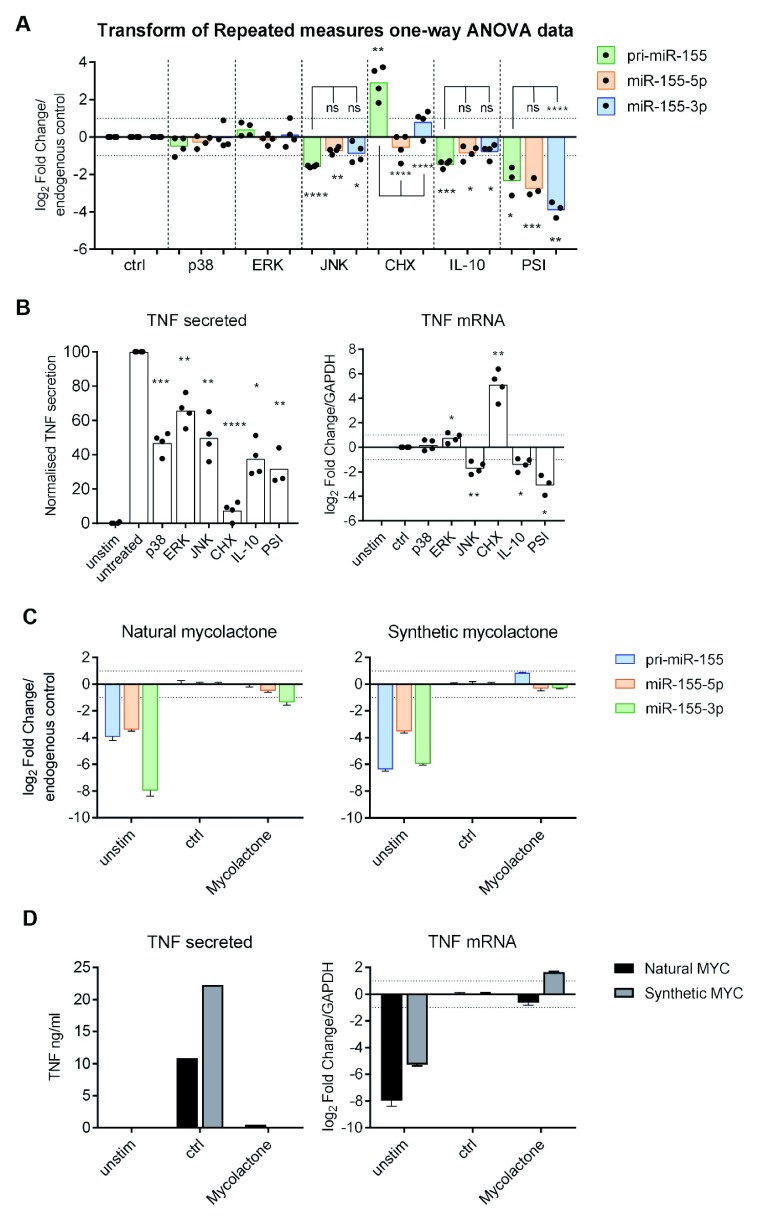
The effect of cell signalling inhibitors, and the *M. ulcerans* exotoxin mycolactone on the expression of miR-155-5p and miR-155-3p. Human 1°MDMs were pre-incubated in the absence or presence of inhibitors/toxin for 1 hr prior to 4 hrs 100 ng/ml LPS stimulation. Total RNA was extracted and cell culture supernatants were collected. Relative changes to the steady state levels of pri-miR-155 (MIR155HG), miR-155-5p, and miR-155-3p ( **A**, **C**) and TNF mRNA ( **B**, **D**) were quantified; the endogenous control for the miRNAs was miR-16-5p, for pri-miR-155 and TNF it was GAPDH. Cytokine secretion was assessed by ELISA. ( **A**, **B**) The effect of different cell signalling inhibitors [SB203580 (p38; 1 µM), PD98059 (ERK, 20 µM), SP600125 (JNK, 10 µM), CHX (inhibitor of translation, 10 µg/ml), and PSI (inhibitor of NFκB pathway, 5 µM)]. Mean±SEM n=3–4; one sample t-test on log _2_ transformed data compared to ctrl (LPS stimulated cells without inhibitors) or one-way ANOVA. *p<0.05, **P<0.01, ***P<0.001, ****P<0.0001. ( **C**, **D**) The effect of mycolactone A/B in different forms (either purified from *M. ulcerans* bacteria “Natural MYC”, or chemically synthesised “Synthetic MYC”). Mean ± error of single donor experiments (see Methods: Statistical analysis).

Where appropriate, data were analysed by either paired t-test ( Figure 1, mRNA/miRNA quantification), one sample t-test on log _2_ transformed data (when compared to control with a relative expression of 1, Figure 2A, B) or ordinary one-way ANOVA with Bonferroni’s multiple comparison test (when comparing the relative changes between pri-miR-155, miR-155-5p and miR-155-3p, or the abundance of mRNAs and miRNAs between equivalent amounts of scrambled vs miRNA specific oligonucleotides). TNF production data was normalised to the control value (100%) then analysed by one sample t-test ( Figure 2B) or ordinary one-way ANOVA with Bonferroni’s multiple comparison test (when comparing the relative secretion of TNF between equivalent amounts of scrambled vs miRNA specific oligonucleotides). All analyses were carried out using Graphpad Prism v7.04.

## Results

### Differential regulation, and differential expression of miR-155-5p and miR-155-3p

A miRNA microarray was performed focussing on the primary response to LPS stimulation (TLR4 activation; 2, 4 and 8 hr) in primary human monocyte-derived macrophages (1°MDMs), and the effect of IL-10 co-incubation. The cells used in the array performed as expected in terms of induction of TNF mRNA and secretion ( Figure 1A), and normalised data from the array appears acceptable ( Figure 1B). The pooled donor approach used has limitations, since statistical analysis of the microarray data cannot be performed. Therefore, observations that are not supported by detailed validation should be considered cautiously. Of the 719 miRNAs examined, 197 were expressed in 1°MDMs (defined as expression greater than background in all six slides) and the common reference sample, representing cells in all tested conditions. The miRNAs exhibited a wide variation in fluorescence intensities in the array (see “List of miRNA expressed in primary human MDMs”, available on OSF ^29^). Over the six slides, the variation in fluorescence intensity of the common reference was 16±4%, suggesting good technical reproducibility. One of the highest expressed miRNAs was miR-16-5p, which was not altered upon exposure of the cells to LPS over the 8-hour time-course of the experiment ( Figure 1C). Raw microarray data are available at the Gene Expression omnibus, accession number GSE125572. All other raw data are available on OSF ^29^.

Only five miRNAs were found to be upregulated >1.5-fold at any time point; miR-155-5p, miR-155-3p, miR-886-5p, miR-886-3p, and miR-147b-3p ( Figure 1C and see “Microarray data for the 197 expressed miRNAs” available on OSF ^29^). None were downregulated, neither were there any changes in response to 4 hrs stimulation with IL-10 alone (range 0.83–1.36-fold, see “Microarray data for the 197 expressed miRNAs” on OSF ^29^). As time increased, more miRNAs were induced. Hence at 2 hrs, only miR-155-5p and miR-155-3p were upregulated >1.5 fold. This increased to four miRNAs (additionally miR-886-5p and miR-886-3p) at 4 hrs, and five (additionally miR-147b-3p) at 8 hrs. The microarray data suggest that IL-10 may both positively and negatively regulate miRNA expression. Compared to 4 hrs with LPS alone, IL-10 increased the expression of miR-766-3p 1.7-fold and miR-146b-3p 2.3-fold, and decreased the expression of miR-155-3p ( Figure 1C, see below). Indeed, miR-155-3p was the most strongly upregulated miRNA at any timepoint.

**Figure 3.**
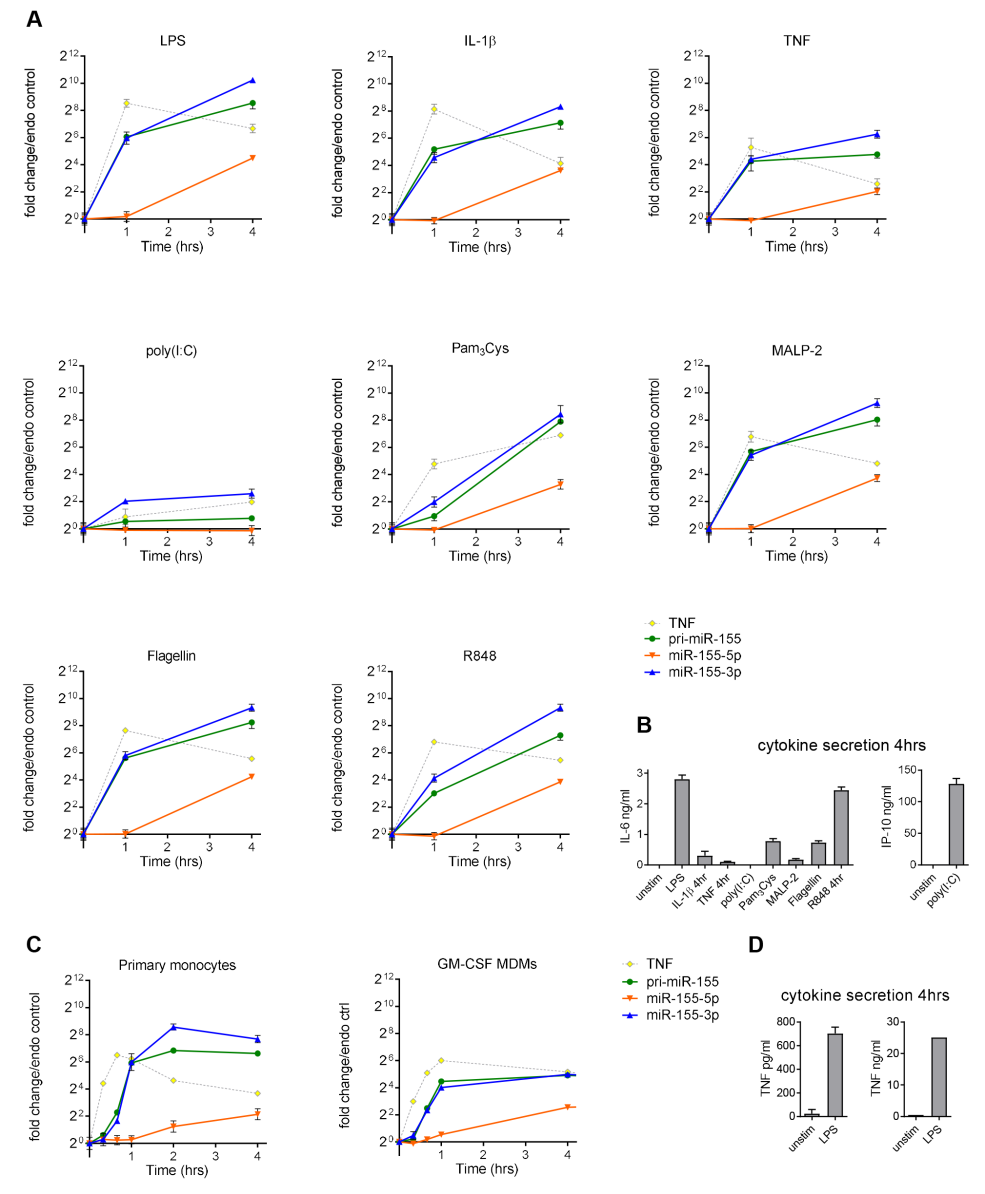
Differential regulation of miR-155-5p and miR-155-3p by other TLR ligands and cytokines, and in other myeloid cells. ( **A**, **B**) Human 1°MDMs were exposed to a range of TLR ligands or cytokines for different time periods and total RNA was extracted and cell culture supernatants were collected. Cytokines were IL-1β and TNF, both at 10 ng/ml. TLR ligands were 10 ng/ml LPS (TLR4); 10 ng/ml, poly(:IC) (TLR3); 20 µg/ml, Pam _3_Cys(TLR1/2); 10 ng/ml, MALP-2 (TLR2/6); 30 ng/ml, Flagellin (TLR5); 10 ng/ml and R848 (TLR7/8); 1 µg/ml. ( **C**, **D**) Either primary monocytes or MDMs differentiated with GM-CSF (as opposed to M-CSF) were exposed to LPS for different time periods and total RNA was extracted. ( **A**, **C**) Relative changes to the steady state levels of TNF mRNA and pri-miR-155, miR-155-5p, and miR-155-3p over time; the endogenous control for the miRNAs was miR-16-5p, for TNF and pri-miR-155 it was GAPDH. Mean±SD of technical triplicates. ( **B**, **D**) Cytokines in supernatants were quantified by ELISA. In **D** the left panel is monocytes, and the right panel macrophages differentiated with GM-CSF.

Validation of the microarray data was achieved by quantifying selected miRNAs by Taqman miRNA qPCR assays in four independent human donors. While no down-regulated miRNA was identified in the array for validation, miR-146a-5p was chosen as it was below the 1.5-fold threshold, despite being in the top 6 increased miRNAs after 4hrs LPS exposure (~1.3-fold). A comparable result was confirmed by PCR ( Figure 3D). Both miR-155-5p and miR-155-3p were chosen for validation of upregulated miRNAs, which was successful since PCR data showed that miR-155-5p levels increased steadily as expected during the timecourse of the experiment ( Figure 1E), miR-155-3p was >100-fold induced at 2 hours, after which the levels began to reduce again ( Figure 1F).

Gene ontology analysis of the miRDB predicted targets of miR-155-5p and miR-155-3p was performed using Panther over-representation test ( Figure 1G). Both microRNAs’ predicted targets were over-represented in genes that participate in the gonadotropin-releasing hormone receptor pathway, IFN-γ signalling and the p53 pathway feedback loops, none of which have an obvious connection to innate immune signalling. However, miR-155-5p targets also appear to be over-representative of the JAK/STAT signalling pathway (PIAS1, PIAS2 and SOCS1) and the p38 MAP kinase pathway (MEF2A, TAB2, MAP3K7, MAP3K10 and RPS6KAS). Interestingly, the TLR signalling pathway was over-represented in the list of potential miR-155-3p targets (MDM4, TEP1, MAPK13, MAPK14).

The unusually large response of miR-155-3p, along with the interesting kinetics, lead to a more detailed investigation of the finding. Changes to the abundance of pri-miR-155 following exposure of macrophages to LPS were monitored alongside miR-155-5p and miR-155-3p in 10 human donors, taking a more detailed look at either early or later timepoints following stimulation (compiled data, Figure 1H). Unfortunately it was not possible to selectively quantify the pre-miR-155 stem-loop precursor. However it is worth noting that previous studies determined that quantitation of pri-miR-155 and pre-miR-155 gives comparable results in leukocytes ^33, 34^. Here, miR-155-3p induction was found to be remarkably rapid, being detected 20 mins after LPS exposure. This seems to correlate with a small ‘dip’ in the levels of pri-miR-155. Subsequently both miR-155-3p and pri-miR-155 induction is remarkably similar, peaking at 2–4 hours and returning to close to resting levels at 24 hours. In stark contrast, miR-155-5p expression rises steadily and continues to rise even as pri-miR-155 levels begin to subside.

These data may suggest that different molecular controls may govern the abundance of miR-155-5p and miR-155-3p independently since their expression correlates poorly. In order to examine the signalling pathways that might be involved in pri-miR-155, miR-155-5p and miR-155-3p induction, small molecule inhibitors of p38, ERK, JNK and the NF-kB pathway were used ( Figure 2A–D) to inhibit LPS-dependent induction. All the inhibitors reduced TNF secretion to different extents, as expected ( Figure 2B). The 4-hour timepoint was chosen as a suitable time at which upregulation of both miR-155-5p and miR-155-3p would be detectable. However, it should be noted that this is somewhat early in the miR-155-5p response (which continues to increase at least to 24hrs (for instance, Figure 1G and 35). Moreover, it is slightly late in the TNF mRNA response, which peaks at ~1 hr ( Figure 3A). This is likely the explanation for the limited effect of p38 and ERK inhibition on TNF mRNA transcription at 4hrs, which could be observed at 1 hr (raw data file 11_005, available on OSF ^29^).

Reduction in LPS-dependent induction of pri-miR-155, miR-155-5p and miR-155-3p by IL-10 was confirmed in these experiments ( Figure 2A), in agreement with previous literature ^8, 33^. However, neither the p38 inhibitor SB203580 nor the ERK inhibitor PD98059 significantly influenced induction at 4 hrs. The JNK inhibitor SP600125 decreased expression of all three molecules similarly by around 50%; however, by far the most profound decrease was with PSI, which decreased pri-miR-155 and miR-155-5p levels about 5-fold, but miR-155-3p levels about 16-fold, a statistically significant difference. In addition, the translation inhibitor cycloheximide was used to investigate whether pri-miR-155 was induced following a primary or secondary response in MDMs. pri-miR-155 was super-induced by cycloheximide, similarly to TNF mRNA in these cells (a well-recognised phenomenon, Figure 2B). However, neither miR-155-3p nor miR-155-5p was significantly changed in these circumstances ( Figure 2A). This data suggests that different pathways may exist that control the abundance of pri-miR-155 compared to either of the mature miR-155 miRNAs, however additional experiments would be required to propose a model.

The *M. ulcerans* exotoxin mycolactone is known to be immunosuppressive and strongly inhibit the production of cytokines, including TNF, from monocytes and macrophages ^36^ due to blockade of Sec61-dependent translocation of proteins into the endoplasmic reticulum ^25^. In order to determine whether LPS-dependent induction of miR-155 was a primary or secondary effect due to alteration of cytokine production, we investigated whether it altered the expression of miR-155 isoforms ( Figure 2C, D). Mycolactone (either purified from the bacteria or chemically synthesised) strongly blocked the production of TNF, whilst having a minimal effect on TNF mRNA abundance ( Figure 2D), as expected ^23^. These experiments only revealed small fluctuations in pri-miR-155, miR-155-5p and miR-155-3p ( Figure 2C) ruling out secondary pathways.

### pri-miR-155, miR-155-5p and miR-155-3p are differentially induced following stimulation by a wide range of proinflammatory cytokines, TLR ligands, and in other primary myeloid cells

In order to examine whether the differential regulation of the two strands of miR-155 derived from pri-miR-155 was unique to LPS (TLR4) stimulation of MDM, a wide variety of different TLR ligands were tested over a timecourse in a single human donor. While further repetition would be need to make firmer conclusions, this data suggested that IL-1β, TNF, Pam _3_Cys (TLR1/2), MALP-2 (TLR2/6), Flagellin (TLR5) and R848 (TLR7/8) are all able induce a broadly equivalent upregulation of steady state pri-miR-155 abundance over the 4hr timecourse of the experiment ( Figure 3A). On the other hand, the TLR3 ligand poly(I:C) only induced a very weak response by pri-miR-155 despite a robust induction of IP-10 cytokine secretion (downstream of type I interferons, Figure 3B).

Furthermore, the response was not limited to MDMs that had been *in vitro* differentiated with M-CSF. Primary monocytes, as well as MDMs differentiated with GM-CSF, also induced pri-miR-155, although the response from GM-CSF MDMs was lower ( Figure 3C) despite a stronger induction of TNF ( Figure 3D). In all cases, perhaps with the exception of poly(I:C), the upregulation of miR-155-3p was more rapid and of a far larger degree than miR-155-5p ( Figure 3). In no cases did upregulation of miR-155-3p outstrip that of pri-miR-155.

### Absolute quantitation of miRNAs using synthetic miR-16-5p, miR-155-5p and miR-155-3p

**Figure 4.**
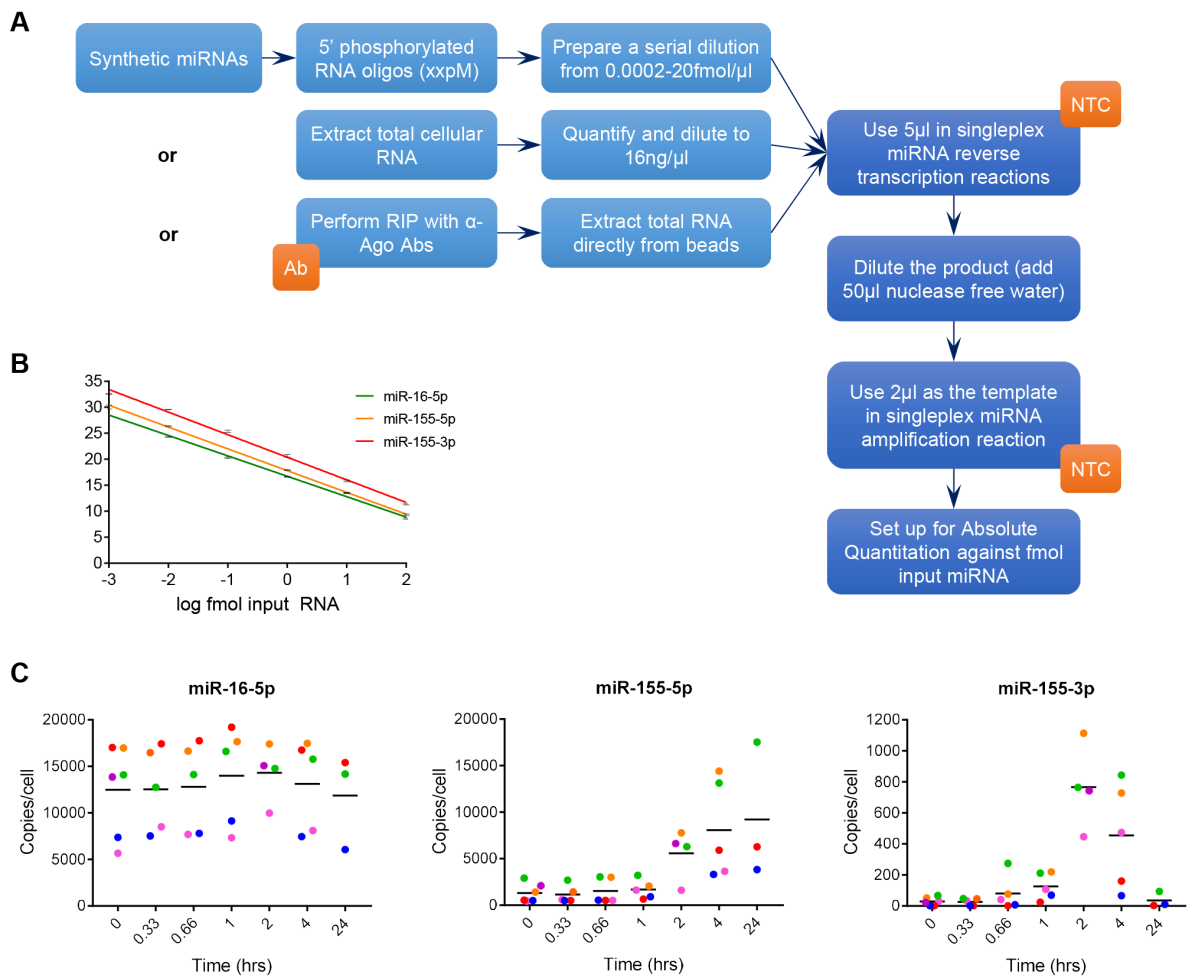
Absolute quantitation of miR-16-5p, miR-155-5p and miR-155-3p in primary human monocyte derived macrophages. ( **A**) Schematic showing the approach for absolute quantitation. ( **B**) Examples of standard curves achieved with the method for each of three miRNAs: miR-16-5p, miR-155-5p and miR-155-3p. Mean±SD of triplicate values are shown. ( **C**) Human 1°MDMs were exposed to 100ng/ml LPS for different time periods. Total RNA was extracted and miRNA abundance was estimated using the absolute quantitation method in singleplex. The donor-to-donor variability of miRNA abundance over time, showing the mean and each donor in a different colour. Note that the prevalence of each miRNA varies independently.

In order to better understand the differential regulation of the two strands of miR-155, an approach was developed that facilitated the absolute quantitation of miRNAs in cells (AQ miRNA, Figure 4A). Since very few specific protocols appear to be available for such an approach, the methodology has been described in detail as a community resource. Examples of the standard curves that resulted from the reverse transcription of synthetic miRNAs are shown in Figure 4B for miR-155-5p, miR-155-3p, and miR-16-5p. This approach routinely resulted in standard curves of high quality, with r ^2^ > 0.99 in all cases over a large input range ( Table 2). The ability of these assays to detect miRNAs differs slightly depending on the miRNA sequence, for instance the detection limit for miR-155-5p and miR-155-3p was 0.001 or 0.0001 fmol, whereas for miR-16-5p it was always 0.0001 fmol. There were also variations in the apparent efficiency for the combined reverse transcription and PCR reactions, which were around 75%, lower than you would expect for qPCR. However, it is important to note that for this is not comparable to the typical PCR efficiency, as it is a two-step assay where the amount of input RNA into reverse transcription was varied. In circumstances where input RNA amounts are, by definition, very low (such as RIP, where RNA is barely detectable by nanodrop, not shown), such a reduction in efficiency should affect the standard and test samples equivalently.

**Table 2.** Performance of standards in the assay. The mean values obtained from n=7 standard curves is shown.

miRNA	r ^2^	Slope	Efficiency (RT&PCR)
miR-16-5p	0.9985	-4.127	75.0%
miR-155-5p *	0.9984	-4.137	75.5%
miR-155-3p *	0.9977	-4.050	77.2%

*On several occasions the lowest standard (0.0001fmol synthetic miRNA) did not give amplification above background; in such cases this point was not considered, increasing the detection limit of the assay to 0.001fmol, and the values are calculated from the remaining points.

### Absolute quantitation of cellular miRNAs reveals the wide difference in abundance of miRNAs

The results of the AQ-miRNA analysis method are shown in Figure 4C and enumerated in Table 3. This showed that there are, on average, ~12,500 copies of miR-16-5p per macrophage (depending on the donor, Figures 4C, summarised in Table 3), whereas resting cells contain ~1,300 copies of miR-155-5p and ~30 copies of miR-155-3p, which rose to an average of ~750 copies/cell at 2 hrs. Hence there is a 400-fold difference in the abundance between miR-16-5p and miR-155-3p in resting cells, but a 16-fold difference after activation. The relative abundance of miR-155-5p and miR-155-3p using this approach was 45-fold in resting cells and 7-fold after 2hrs of LPS stimulation.

**Table 3.** Copy-numbers per cell of different miRNAs obtained using the absolute quantitation approach. Compiled data from n=4–6 human donors is shown. *For some donors, this was below the lowest detectable standard in the assay.

miRNA	Condition	Total copies/cell	
		Mean ± SEM	Range
**miR-16-5p**	resting	12,496±1,984	5,653–17,029
	2hrs LPS	14,306±1,561	9,969-17,048
	24hrs LPS	11,880±2,935	6,052–15,406
**hsa-miR-** **155-5p**	resting	1,315±417	448–2,915
	2hrs LPS	5,578±1,361	1,609-7,777
	24hrs LPS	9,203±4,216	3,820–17,517
**hsa-miR-** **155-3p**	resting	29±11	1.6 *–67
	2hrs LPS	767±137	446–1,134
	24hrs LPS	35±29	3 *–94

*For some donors, this was below the lowest detectable standard in the assay.

It seems likely that the variation seen here reflects truly wide donor-to-donor variability. Transforming the fmol/80ng readout of the AQ-miRNA assay into copies/cell also makes a number of assumptions that may contribute to this variation between experiments, such as similar RNA extraction efficiency and cell counting accuracy. However, the prevalence of each miRNA varies independently (for example, the donor displayed in red in Figure 4C had the highest miR-16-5p, intermediate miR-155-5p levels and the lowest miR-155-3p abundance).

### Despite its low abundance, miR-155-3p associates with the RISC

One explanation for the low numbers of miR-155-3p molecules per cell is that they are simply an artefact of the large increase in pri-miR-155 and associated processing of pre-miR-155-5p into miR-155-5p. Such processing would presumably release the passenger strand, which is not incorporated into the RISC, and this might be detectable for a short period before it is degraded. Alternatively, the pre-miRs may be actively processed to result in miR-155-3p loading in the RISC where they have the potential to carry out biological functions.

**Figure 5.**
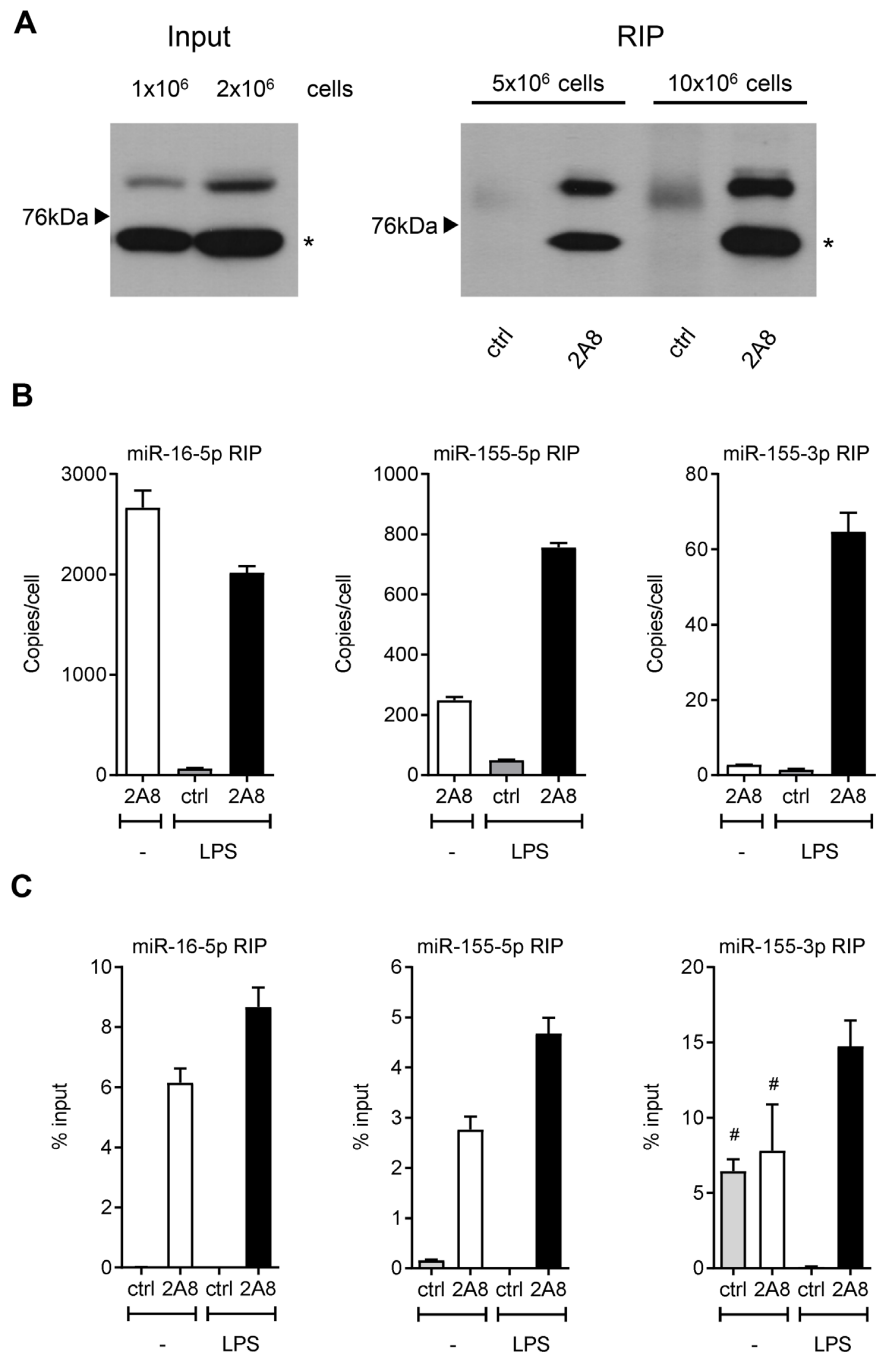
miR-16-5p, miR-155-5p and miR-155-3p may all be loaded into the RISC in primary human monocyte derived macrophages. ( **A**– **C**) The RISC complex in human 1°MDM was immunoprecipitated using antibodies that recognise Argonaute proteins (2A8) or control sera (ctrl). All data is representative of n=3 independent donors. ( **A**) Western blot analysis of input protein (left panel) or eluted protein (right panel). Note that the antibodies cross-react with a protein called Radixin (*). ( **B**) miRNAs loaded into the RISC were isolated by RIP using 2A8 and control antibodies. RNA isolated from the RISC were subject to absolute quantitation. Mean±SD of triplicate values. ( **C**) miRNAs loaded into the RISC were isolated by RIP using 2A8 and control antibodies. RNA isolated from the RISC were quantified as a proportion of those in the input. Mean±SD of duplicate values. #: both the input and RIP samples for miR-155-3p had Ct values of 35-36, at the detection limit of the PCR.

In order to examine this, the incorporation of miRNAs into the RISC was estimated by enriching them using RIP and antibodies that recognise human Argonaute proteins. Control experiments showed that the approach successfully immunoprecipitated the proteins ( Figure 5A); as previously reported there was some cross-reactivity to a non-relevant protein called Radixin ^30^. Using this approach, several thousand copies/cell of miR-16-5p were associated with the RISC in both unstimulated and stimulated cells ( Figure 5B). Fewer copies of miR-155-5p were detected in this way; several hundred copies/cell in resting cells, which increased in stimulated cells ( Figure 5B). Note that at this timepoint, there had only been a 3-fold increase in total cellular miR-155-5p (donor shown in pale blue in Figure 4C). Importantly, although miR-155-3p could not be detected within the RISC above background levels in resting cells, following LPS stimulation for 2hrs (the peak of miR-155-3p detection in cells) it could be recovered with similar efficiency ( Figure 5B). This was also true when an alternative method for estimating the proportion of miRNAs incorporated into the RISC was used ( Figure 5C).

### Technical challenges and limitations of assessing miRNA targets in primary human MDM

**Figure 6.**
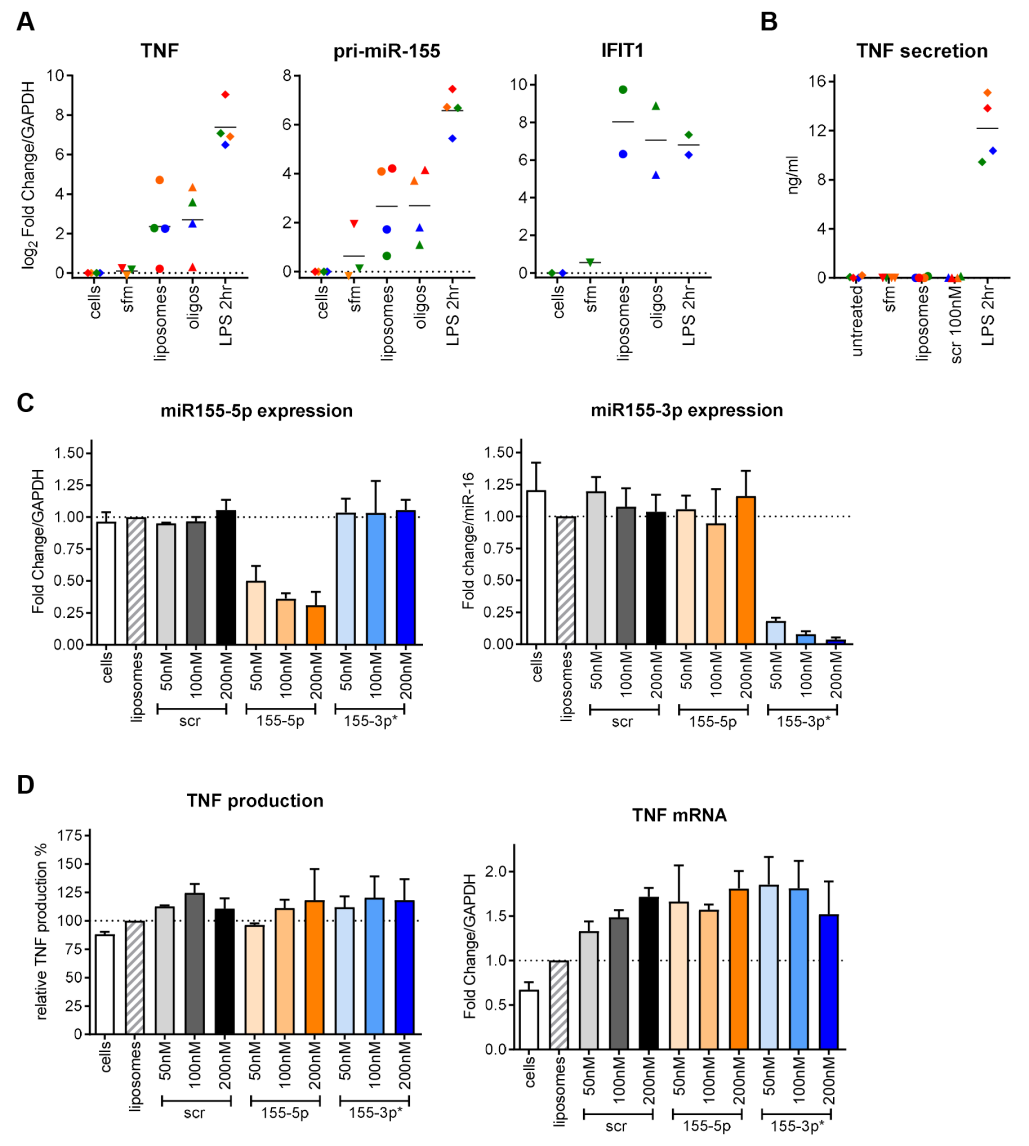
No effect of miR-155-5p or miR-155-3p inhibition on the production of TNF by primary human MDMs. Human 1°MDMs were transfected with miRcury LNA miRNA knockdown probes (either the negative control Scramble-miR, scr, or miR-155-5p or miR-155-3p) using Dharmafect 1. Duplicate 2 hr transfections were performed and one set of cells were stimulated with LPS for 2 hrs, while the unstimulated rested for an additional 2 hrs. Supernatants were collected for the measurement of secreted cytokines and total RNA was extracted. ( **A**) Abundance of TNF and IFIT1 mRNA and pri-miR-155 by fold-change in unstimulated control cells, compared to cells alone stimulated with LPS (comparator; GAPDH). Each dot is the mean of technical triplicates from qPCR. ( **B**) TNF secretion from unstimulated control cells, compared to cells alone stimulated with 100 ng/ml LPS. Each dot is the mean of technical duplicate from ELISA. ( **C**) Efficiency of “knockdown” was assessed by relative abundance of miRNAs in LPs-stimulated cells (comparator; miR-16-5p). Mean±SEM, n=2–4. ( **D**) Relative abundance of TNF mRNA and secreted protein in the LPS-stimulated cells. Mean±SEM, n=2–4.

The primary focus of this study was to attempt to identify miRNAs that could regulate the primary immune response. The rapid induction of miR-155-3p seemed to potentially in line with this hypothesis, considering that the TLR signalling pathway was over-represented in Gene Ontology analysis ( Figure 1G). A 7-mer match for miR-155-3p is predicted in the 3’ UTR of the human TNF mRNA in the Targetscan database , although this is not well conserved amongst mammals. LPS/TLR4-dependent signalling in 1°MDMs induces TNF mRNA within 20mins and TNF secretion within 1hr. To analyse this further, inhibition of miRNAs was achieved using LNA knockdown oligonucleotides that form stable duplexes with both miR-155-5p and miR-155-3p ( Figure 6). A timepoint of 2 hrs was chosen for this experiment, taking into account the kinetics of the different induced mRNAs being studied; hence, at 2 hrs induction of TNF mRNA, pri-miR-155 and miR-155-3p are all near their peak and miR-155-5p induction can be detected. A significant challenge here was that the liposomes used for transfection have profound effects on the signalling pathways within cells even in the absence of exogenous DNA. For instance, unstimulated 1°MDMs that had been exposed to liposomes in serum-free medium (but not serum-free medium alone) showed increases in TNF mRNA and/or pri-miR-155 ( Figure 6A), without the production of TNF protein into the media ( Figure 6B). This may be due to activation of a type I interferon response since, in the two donors studied, IFIT1 expression was also upregulated by liposomes ( Figure 6A). In 1°MDMs that were subsequently stimulated with LPS, these differences were less profound ( Figure 6D), and so while this meant it is not technically meaningful to report the effect of inhibition on fold induction of effectors, it is possible to report some outcomes of this experiment with reasonable confidence, by examining only the data from LPS-stimulated cells, and using the liposome-treated cells as the control/comparator group.

Good dose-dependent knockdown of both miRNAs was achieved by this method ( Figure 6C), with the maximum effect seen at a 200 nM oligonucleotide concentration. However, no effect of inhibiting the function of either miRNA could be seen on the production of TNF into cell culture supernatants or on TNF mRNA.

Raw data for all experiments are available

## Discussion

This study investigated changes to miRNA abundance in the primary response of human 1°MDMs to LPS. The time periods chose were deliberately short in order to focus on primary rather than secondary effects; for instance the plethora of cytokines induced by activation of this inflammatory pathway also have potent cellular effects themselves. A key finding is that very few miRNAs are dynamically regulated within this time frame. No miRNAs decreased in abundance in any of the conditions tested, and IL-10 alone had no discernible effect on miRNA expression. Only five out of 197 expressed miRNAs showed a >1.5-fold change at any time point. Since it is now thought that both hsa-miR-886-5p and hsa-miR-886-3p are fragments of Vault RNA ^37^, rather than true miRNAs (and have subsequently been withdrawn from miRbase), the number may be even lower.

The most dynamically regulated miRNA in this study was miR-155-3p, the partner strand of miR-155-5p within highly conserved microRNA, miR-155. It is undoubted that miR-155 (MIR155HG/BIC) and miR-155-5p have important roles in the immune response, including both the innate and adaptive arms (extensively reviewed in 8). In summary, using genetic knockout mice lacking BIC, miR-155 has been shown to be indispensable for the B cell maturation, migration, interaction with T cells and antibody production ^8^. Furthermore, it has been shown to regulate T cell differentiation including promoting Th1, Th17 and Tfh development while limiting Th2. Finally, miR-155 is required for the development of Tregs and antiviral CD8 T cell responses. 

Focussing on the innate immune functions of miR-155, it is well-established that pri-miR-155 and miR-155-5p are induced by pro-inflammatory stimuli and that this can be regulated by negative regulators such as IL-10, TGF-β, glucocorticoids and Resolvin D1 ^8, 33, 35, 38– 41^. Direct cellular targets for miR-155-5p reported in monocytes, macrophages and dendritic cells include SHIP1 [INPP5D; a phosphatase that hydrolyses the 5’ phosphate from phosphatidylinositol (3,4,5)-trisphosphate and inositol-1,3,4,5-tetrakisphosphate and hence acts as a negative regulator of TLR/PI3/Akt kinase pathway signalling] and SOCS1 (a negative regulator of cytokine and TLR-mediated immune responses). In macrophages, other direct targets include SMAD2 (part of the TGFβ signalling cascade), BCL6 (a transcription factor that negatively regulates NFκB signalling in macrophages) and LXRα [NR1H3, a nuclear receptor family transcription factor that inhibits inflammatory responses in macrophages and promotes anti-inflammatory markers such as Arginase 1 (ARG1)] ^8^. These targets are unified by promoting regulatory or negative effects on inflammatory signalling, and hence inhibition of miR-155-5p induction (either by genetic knockout or knockdown) has been reported to enhance TNF secretion in response to LPS ^21, 22^. In addition to an indirect effect, via the direct targets listed above, miR-155-5p was reported to directly stablise the TNF mRNA ^22^. However, these studies measured TNF responses at later timepoints after LPS stimulation than the current report (2hrs vs. 18–24hrs in other studies), suggesting that miR-155-5p has a greater impact later in the response when its expression has increased further and/or secondary responses have had time to develop. In addition, some studies used cells that had a complete knockout of miR-155, hence may have a fundamentally different phenotype to wild type primary cells where the miRNA function is blocked.

Fewer studies have examined the role of miR-155-3p, never-the-less a functional role for miR-155-3p is supported by studies in which cellular targets have been described. In plasmacytoid dendritic cells and trophoblasts it is a pro-inflammatory, augmenting type I interferon expression by suppressing IRAK-M (IRAK3) early after TLR7 activation of the cells ^11, 13^. IRAK-M is a negative regulator of TLR signalling via Myd88 whose expression is restricted to myeloid cells ^42^, including the macrophages that were the focus of the current work. By restricting its expression transiently during the cell activation process via miR-155-3p, the inflammatory burst from the cells would be enhanced for a limited period, although this was not directly studied. Other reported targets include NKIRAS and PTEN in trophoblasts ^13^, two Hsp40 genes (Dnaja2 and Dnajb1) controlling murine Th17 differentiation ^16^, MEF2C during cardiogenesis from embryonic stem cells ^17^, CREBRF during the hypoxia-induced IL-6 response in glioblastoma ^18^, and lymphotoxin-beta (LT-β) a positive regulator of non-canonical NF-kB signalling in MCL where it has tumour suppressing properties ^19^.

While most of these targets of miR-155-5p and miR-155-3p are listed in databases of predicted targets such as miRDB and Targetscan, only SOCS1 appeared in a statistically over-represented pathway in Gene Ontology analysis suggesting that such an approach might miss biologically relevant targets. TNF is listed as a predicted direct target of miR-155-3p, albeit poorly-conserved amongst mammals. However, TNF secretion was not affected by miR-155-3p inhibition even when examined at peak induction. No other functional analysis was performed in the current study.

The differences in relative induction demonstrated for miR-155-5p and miR-155-3p in LPS/TLR4-stimulated macrophages is in line with a previous report that studied the TLR7 (R837, also known as Imiquimod) response in plasmacytoid dendritic cells, with similar kinetic and amplitude characteristics ^11^. These authors proposed a model whereby the differential regulation of the miRNAs was ascribed to KH-type splicing regulatory protein (KHSRP), which promoted miR-155-5p maturation but inhibited miR-155-3p production ^11^. Further work is needed to determine whether the same KHSRP-dependent mechanism also controls isomer abundance in the LPS response in macrophages, but this is likely since the NFκB pathway is activity by both TLR4 and TLR7/8. Furthermore, other have also shown that KHSRP binds to the terminal loop of miR-155 and enhances maturation of miR-155-5p during the LPS response in murine bone marrow-derived macrophages, although miR-155-3p was not quantified in this study ^43^.

Other mechanisms might also explain the phenotype observed, such as changes to the stability and/or degradation rates of the different isoforms during the TLR response. Recently, miR-155-5p was shown to have a half-life of 10.5hrs, considered to be “fast” decaying miRNA ^44^, although it is not yet known how TLR-dependent stimulation alters these dynamics. Measuring the precursor pre-miR-155 levels during the response using an approach that would not also detect pri-miR-155 is considered essential to the future studies but was not done here, or in previous investigations ^11, 43^.

Such regulation of the two mature miRs that can derive from the single pre-miR, so-called “arm switching/selection” ^4^ is of increasingly intense research study. For instance, in a long time-course study of IFNγ activation of melanoma cells, 4 of the 10 highest regulated miRNAs were ‘star’ strands (miR-424-3p, miR-29b-1-5p, miR-27a-5p and miR23a-5p; current miRbase annotations), that were induced where the partner strands were not ^45^. Arm selection in miR-193a ^46^ and miR-324 ^47, 48^ also play important roles in cancer. Molecular mechanisms have remained elusive so far, although in at least one case this may be driven by temperature. Polta and colleagues identified three miRNAs, previously denoted as passenger strands (hsa-miR-92a-1-5, hsa-miR-27b-5p, and hsa-miR-1260a), that had altered expression when the temperature varied between 32–39.5°C, and that together regulated the expression of PKCα ^49^.

Experiments with chemical inhibitors of different signalling pathways showed that, in line with the findings of others, the upregulation of miR-155-5p was reduced by IL-10, as well as by inhibition of the JNK/AP1 and NFκB pathways (in the latter case by inhibiting proteasomal degradation of IκBα with PSI) ^33, 35, 38– 41^. This explains the much lower induction of pri-miR-155 in poly(I:C) treated cells since TLR3 is not functionally linked to NFκB activation in human 1°MDMs ^50^, hence the small induction might be JNK/AP1-dependent. ERK and p38 inhibition had no effect, again in line with previous findings ^35, 38^. Interestingly, the relationship between pri-miR-155 and miR-155-3p abundance was maintained during JNK and IL-10 treatment, but lost in the presence of PSI where NFκB activation resulted in a greater decrease in mature miRNA-155-3p than the pri-miR-155 precursor. This discrepancy might be a potential starting point for further investigations to establish the underlying mechanism of the phenotypes observed. Finally, it is well established that CHX causes super-induction of LPS-dependent TNF mRNA ^51^, as is the case in the current work. The mechanism is not known; however, while it is possible that a similar mechanism operates for pri-miR-155, there is no evidence to support the need for *de novo* protein synthesis in the production of miR-155-5p ^52^.

Despite miR-155-3p undergoing the greatest change in expression level of any miRNA in primary human MDMs, the absolute levels of this miRNA are very low. In resting cells, the abundance of miR-155-3p was close to the detection limit of the qPCR assays. For a short, transient period after LPS challenge the number of copies/cell of this miRNA become in the same order as resting levels of miR-155-5p. Furthermore miR-155-3p could be recovered from the RISC at this timepoint, suggesting that it may have biological function despite its low abundance. The use of the AQ miRNA approach is therefore a vital addition to fully understand the biological significance of such data. The lower limit for miRNA abundance required for biological activity is not known, although the Targetscan database defines it as 1,000 copies/cell. None of the papers reporting functional analysis for miR-155-3p described above provided data on the absolute levels of this miRNA in their particular model. Such data would potentially provide fine-tuning of this proposed threshold, especially since target validation for miRNAs frequently involves over-expressing pre-miRs to non-physiological levels,

In conclusion, this work provides a detailed description of the induction of miR-155-3p in human 1°MDMs and other myeloid cells by a range of inflammatory stimuli including LPS. At the peak of its abundance, miR-155-3p is transiently present at close to 1,000 copies/cell, and can be recovered from the RISC. Together with the functional analysis done by others, this lends weight to the assertion that miR-155-3p might have biological function in a restricted range of circumstances where it is induced. The limitations of the current study are the absence of quantification of the pre-miR-155 precursor, the low abundance and wide range of the miR-155-3p response between individual donors, and the lack of functional experiments. Some of the limitations are technical, resulting from the speed of the miR-155-3p response, the exquisite sensitivity of 1°MDMs to PAMPs, apparently including the liposomes used to transfect oligonucleotide inhibitors into the cells. More work needs to be done to determine if miR-155-3p has a role in inflammation or the immune response. Future studies of miR-155-3p should include absolute quantitation alongside relative quantitation, analysis of precursor pre-miR-155 and functional analysis.

## Data availability

### Underlying data

Microarray data (title: microRNA responses of LPS and IL-10 stimulated primary human monocyte-derived macrophages over a short time-course) are available at GEO: accession number GSE125572.

Open Science Framework: Transient up-regulation of miR-155-3p by lipopolysaccharide in primary human monocyte-derived macrophages results in RISC incorporation but does not alter TNF expression. https://doi.org/10.17605/OSF.IO/KUBCX ^29^.

The following underlying data files are available:

- Experiment 11_089 Effect of inhibitors on mRNA and miRNA.xlsx
- Experiment 11_005 Effect of inhibitors on mRNA and miRNA.xlsx
- Experiment 11_023 and 11_027 comparison of GM- vs M-CSF-differentiated macrophages.xlsx
- Experiment 11_031 AQ Absolute quantification of miRNAs.xlsx
- Experiment 11_055 Effect of inhibitors on mRNA and miRNA.xlsx
- Experiment 11_059 RIP of miRNAs and mRNAs.xlsx
- Experiment 11_083 RIP of msRNAs and mRNAs.xlsx
- Experiment 11_111 Effect of inhibitors on mRNA and miRNA.xlsx
- Experiment 5_125 delta delta Ct Timecourse of LPS induction with and without mycolactone or IL-10.xls
- Experiment 5_149 whole blot for Ago proteins with 2A8.tif
- Experiment 5_161 AQ and RQ RIP of miRNAs.xls
- Experiment 5_171 AQ and ddCt RIP of miRNAs.xls
- Experiment 5_181 Timcourse of miRNA and mRNA induction.xls
- Experiment 5_195 Induction of mRNA and miRNA by different TLR ligands.xls
- Experiment 5_199 mRNA and miRNA induction by LPS in monocytes vs macrophages.xls
- Experiments 5_155 & 5_161 whole blot for Ago proteins with 2A8.tif

### Extended data

Open Science Framework: Transient up-regulation of miR-155-3p by lipopolysaccharide in primary human monocyte-derived macrophages results in RISC incorporation but does not alter TNF expression. https://doi.org/10.17605/OSF.IO/KUBCX ^29^.

The following extended data files are available:

- Experiment log showing the datasets used for each figure of the manuscript.xlsx
- List of miRNA expressed in primary human MDMs.xlsx
- Microarray data for the 197 expressed miRNAs.xlsx

Data are available under the terms of the Creative Commons Attribution 4.0 International license (CC-BY 4.0).
