# Supplementary material for: Transient up-regulation of miR-155-3p by lipopolysaccharide in primary human monocyte-derived macrophages results in RISC incorporation but does not alter TNF expression
Source: Wellcome Open Res. 2019 Oct 3;4:43. Originally published 2019 Mar 6. [Version 2] doi: 10.12688/wellcomeopenres.15065.2 (PMC6790912; doi:10.12688/wellcomeopenres.15065.2)
Supplement: Peer review history

# Peer review: Reviewer response for version 2

**DOI:** 10.21956/wellcomeopenres.16915.r36639

**Published:** 2019-11-26

**Authors:** Jochen Imig

**Affiliations:** 1 Institute of Pharmaceutical Sciences, ETH Zurich, Zurich, Switzerland

All the reviewers comments in the revised manuscript are sufficiently addressed. There are no further issues regarding the manuscript to be indexed in Wellcome Open Research.

I confirm that I have read this submission and believe that I have an appropriate level of expertise to confirm that it is of an acceptable scientific standard.

# Peer review: Reviewer response for version 2

**DOI:** 10.21956/wellcomeopenres.16915.r36646

**Published:** 2019-10-21

**Authors:** Suvendra N. Bhattacharyya, Avijit Goswami

**Affiliations:** 1 RNA Biology Research Laboratory, CSIR-Indian Institute of Chemical Biology, Kolkata, India

We have gone through the changes introduced and are happy to approve it.

We confirm that we have read this submission and believe that we have an appropriate level of expertise to confirm that it is of an acceptable scientific standard.

# Peer review: Reviewer response for version 2

**DOI:** 10.21956/wellcomeopenres.16915.r36638

**Published:** 2019-10-10

**Authors:** Gracjan Michlewski

**Affiliations:** 1 Division of Infection and Pathway Medicine, University of Edinburgh, Edinburgh, UK; 2 Zhejiang University - University of Edinburgh Institute, Haining, China

I am convinced by the response to my original review. Moreover, authors did a good job of by toning down their statements as well as citing appropriate literature.

 I am unconvinced that it is not be possible to distinguish pro-miR-155 from pre-miR-155. One simple solution (which we routinely use in the lab) is to resolve total RNA on a desaturating gel, cut out the fraction corresponding to the 50-100nt long pre-miRNA and quantify with standard SYBR green approach. Finally, pri-miR-155 can be easily distinguished from pre-miR-155 by using primers that lie outside of the pre-miR-155 sequence.

I confirm that I have read this submission and believe that I have an appropriate level of expertise to confirm that it is of an acceptable scientific standard.

# Response

**Published:** 2019-10-10

**Authors:** Rachel Simmonds

**Affiliations:** University of Surrey, UK

Many thanks for taking the time to review the revised version of the manuscript.

On a technical note, the papers that I referred to in my previous comments where pri-miRNA and pre-miRNA levels were both measured, these did not perform any additional enrichment for small RNAs but used total RNA. Indeed, your method would provide the necessary discrimination.

# Peer review: Reviewer response for version 1

**DOI:** 10.21956/wellcomeopenres.16435.r35299

**Published:** 2019-05-15

**Authors:** Suvendra N. Bhattacharyya, Avijit Goswami

**Affiliations:** 1 RNA Biology Research Laboratory, CSIR-Indian Institute of Chemical Biology, Kolkata, India

In this communication “Transient up-regulation of miR-155-3p by lipopolysaccharide in primary human monocyte-derived macrophages results in RISC incorporation but does not alter TNF expression”, the author tried to identify an unconventional miR-155-3p to be transiently upregulated during early hours of LPS stimulation in primary monocyte derived macrophages. The author reported only 5 miRNAs that happens to be upregulated during initial LPS treatment. It is quite commendable to observe the intent of the author to quantify the absolute number of miRNA copies/ cell. miR-155-5p and miR-146a are the two very well-studied miRNAs that has been investigated in context to immune response. The role of miR-155-3p is yet to be substantiated along with identification of its targets. Some issues regarding this study have been discussed below.

1. miR-155-5p is the predominant strand that has been shown to have a copy number of around 1315±417 in resting phase and 9203±4216 during 24 hours of LPS stimulation. Copy number of miR-155-3p was shown for resting, 2 hours and 24 hours of LPS stimulation. Strangely, the author missed out mentioning the copy number of miR-155-5p at 2 hours of LPS treatment. Absolute quantification of miR-155-5p at 2 hours of LPS treated must be provided to comment on miR-155-3p as “most strongly upregulated miRNA at any timepoint”.
2. The author mentioned with reference from TargetScan Database miR-155-3p is designated as “not so confidently identified miRNA” due to its relatively low abundance of about 1000 copies/cell to be considered biologically functional. The author reported an average 750 copies of miR-155-3p per cell (which the author also considers as an issue). Can this be considered biologically functional? Even if there is a 400 fold increase in the number, it still doesn’t qualify for the 1000 copy cut off.
3. miR-155-5p has a profound effect having previously validated targets, can miR-155-3p shadow the effect of miR-155-5p?
4. Processing of pre-miR-155 and half-life of miR-155-3p could be another possible reason for increased number of miR-155-3p, the author should check the precursor level.
5. Even if there is RISC incorporation of miR-155-3p, the copy number is almost 100-times less than miR-155-5p.

Overall, although the idea is interesting, the abundance of miR-155-3p is a real concern.

We confirm that we have read this submission and believe that we have an appropriate level of expertise to confirm that it is of an acceptable scientific standard, however we have significant reservations, as outlined above.

# Response

**Published:** 2019-09-06

**Authors:** Rachel Simmonds

**Affiliations:** University of Surrey, UK

*Thank you for taking the time to review my manuscript. As mentioned in the introduction, it reports historical data (several years past). While I agree that the additional experiments suggested would give greater insight to the data, there is no prospect of performing them as the lab where the work was done no longer exists (please see the acknowledgements). Hence this submission to WOR, whose unique approach of seeking to publish “results worth sharing” rather than complete stories has given a route to release the data and methodology within, that otherwise would have remained unpublished.*

*I have therefore modified the text to reflect your concerns and the additional/alternative approaches suggested, and have looked closely at the manuscript overall to ensure that the conclusions are not overstated on the basis of the available data.*

miR-155-5p is the predominant strand that has been shown to have a copy number of around 1315±417 in resting phase and 9203±4216 during 24 hours of LPS stimulation. Copy number of miR-155-3p was shown for resting, 2 hours and 24 hours of LPS stimulation. Strangely, the author missed out mentioning the copy number of miR-155-5p at 2 hours of LPS treatment. Absolute quantification of miR-155-5p at 2 hours of LPS treated must be provided to comment on miR-155-3p as “most strongly upregulated miRNA at any timepoint”.

*This data is represented in Figure 4C, but I agree that only showing the 2 hours data for miR-155-3p in Table 3 was an omission. This has now been corrected.*

The author mentioned with reference from TargetScan Database miR-155-3p is designated as “not so confidently identified miRNA” due to its relatively low abundance of about 1000 copies/cell to be considered biologically functional. The author reported an average 750 copies of miR-155-3p per cell (which the author also considers as an issue). Can this be considered biologically functional? Even if there is a 400 fold increase in the number, it still doesn’t qualify for the 1000 copy cut off.

miR-155-5p has a profound effect having previously validated targets, can miR-155-3p shadow the effect of miR-155-5p?

*Indeed, I complete agree with both these important points. Others have proposed and published functional roles for miR-155-3p based upon relative expression and without performing absolute quantification (sometimes based upon massive over-expression of miRNA by transfecting in miRNA mimics that would be present at far higher levels than upon cellular stimulation). Moreover, this quantification places miR-155-3p at the limit of biological function under only a time limited circumstance, albeit that the miRNA can be found within the RISC, and therefore potential of binding to and regulating targets. The new paragraph at the end of the discussion is intended to bring these issues out more clearly.*

Processing of pre-miR-155 and half-life of miR-155-3p could be another possible reason for increased number of miR-155-3p, the author should check the precursor level.

*I agree that analysing the abundance of the pre-miR-155 hairpin would be ideal, and I have looked again to see if this would be possible and concluded the following:*

- *No commercial suppliers make a validated assay for pre-miR-155*
- *Previous papers (eg O’Connell PNAS 2007) find that pre-miR-155 is barely detectable at any timepoint before or during LPS stimulation of BMDM by Northern blot*
- *Very few papers report quantifying pre-miR-155, and in call cases they use in-house SYBR-based PCR that cannot technically discriminate between pre-miR-155 and pri-miR-155 (PCR of the stemloop sequence would amplify both the pre-miR and pri-miR) *
- *In those papers that do directly compare pri-miR and pre-miR abundance for miR-155, they show that the assays give broadly comparable results (not surprising given the above)*
- *Other studies of miR-155-5p and -3p biogenesis (eg Zhou et al, Blood 2010, Ruggiero et al FEBS Lett 2019) also use pri-miR-155 assays to quantify the precursors, as I did.*

 
*Given that it is not possible to generate further samples for alternate approaches such as Northern blotting, I have made modifications to the results and discussion in light of the above. Furthermore, taking the approach of Zhou et al I have relabelled “BIC” as “pri-miR-155” in all figures and text, to clearly indicate the precursors that have been quantified.*

*Examining the half-life of miR-155-3p would also have been an interesting experiment, especially since miR-155-5p was recently shown to have a half-life of 10.5hrs (Marzi et al, Genome Res 2016 p554). Such notions of miRNA degradation and stability are only recently emerging and tackling this issue is out of the scope of the current manuscript. However, a discussion of this alternative explanation of the data is now given in the results and discussion.*

*As mentioned above, setting up additional experiments is not possible, hence the release of this data via Wellcome Open Research rather than a conventional publishing platform.*

# Peer review: Reviewer response for version 1

**DOI:** 10.21956/wellcomeopenres.16435.r35298

**Published:** 2019-05-08

**Authors:** Jochen Imig

**Affiliations:** 1 Institute of Pharmaceutical Sciences, ETH Zurich, Zurich, Switzerland

The author uses an outdated microarray technology to characterize the miRNAs involved in primary immune responses to LPS in human monocyte derived macrophages (MDMs). Only a surprisingly small fraction of actively expressed miRNAs (5 of 197 out of a total 719 on the array) were induced at least 1.5 fold, with miR-155-3p being the strongest one. This stands in contrast to previous reports that the other dominant strand (5p) provided the biologically relevant miRNA. The bulk of the manuscript relies on *in vitro *simulations followed by time course analysis of relative and absolute expression of different readouts such as TNF, the BIC locus or the two miR-155 strands (5p and 3p). Although suppression of miR-155 had no effect on TNF levels, the author suggests that differential arm selection could play an important role in controlling immune responses. Lacking functional experiments, as well as no data to propose a mechanistic model, the author proposes a method for absolute quantification of miRNAs using oligo templates to generate a standard curve. Using this method, it is shown that A) absolute quantification of specific miRNAs is feasible and B) miR-155-5p levels exceed miR-155-3p by approximately an order of magnitude. The author should address a few points, as follows:

1. The microarray data should be validated with an orthogonal approach, such as a panel of selected miRNA (up- down-regulated and unchanged) by Taq-Man qRT-PCR or miRNA-Seq of at least 3 healthy individual donors to show the technical validity and reliability of the miRNA expression and candidate selection thereof.
2. No functional readouts are included for any of the experiments (for example, phagocytic activity or M1/M2 polarization) upon miR-155-3p OE or knockdown.
3. Although it is claimed that miR-155 is one of the most well-known miRNA involved in immune responses, the data in the paper suggests that at least in MDM, it does not play an important role in TNF secretion. This point needs further clarification. 

Specific changes

**Introduction** - Paragraph 2

 Should read "BIC knockout causes defects in germinal center formation and Ig class switching, leading to immunodeficiency".

**Methods**

 Inhibition of miRNAs

 Last sentence needs rephrasing or clarification "since pilot experiments showed that extending the recovery time to significantly reduced the efficiency of inhibition below a meaningful level."

**Results**

 Figure 2C) Unclear why the author uses a crude extract of the toxin, when results from a pure synthetic version are already presented.

 Figure 3C) This figure should read "Primary monocytes" instead of "Primary monocytes". It is also unclear why the author chooses a different cytokine for MDM production.

**Discussion**

 Being a sole author, it is understandable that there are physical limitations to the number of assays that can be performed on primary cells. But this is an obvious condition, making it unnecessary to reiterate it in the discussion section. The connection between miR-155 and immune activation in the model system used still remains unclear and needs further literature to support a model.

I confirm that I have read this submission and believe that I have an appropriate level of expertise to confirm that it is of an acceptable scientific standard, however I have significant reservations, as outlined above.

# Response

**Published:** 2019-09-06

**Authors:** Rachel Simmonds

**Affiliations:** University of Surrey, UK

*Thank you for taking the time to review my manuscript. As mentioned in the introduction, it reports historical data (several years past), and the “outdated” technology current at the time the experiment was performed. This submission to WOR, whose unique approach of seeking to publish “results worth sharing” rather than complete stories has given a route to release the data and methodology within, that otherwise would have remained unpublished.*

The microarray data should be validated with an orthogonal approach, such as a panel of selected miRNA (up- down-regulated and unchanged) by Taq-Man qRT-PCR or miRNA-Seq of at least 3 healthy individual donors to show the technical validity and reliability of the miRNA expression and candidate selection thereof.

*The manuscript already included orthogonal validation of two upregulated miRNAs (miR-155-5p and miR-155-3p). Two other upregulated miRNAs (miR-886-5p and -3p) were also validated but I prefer not to include this data in the manuscript as miR-886 is no longer in miRbase. No miRNAs were downregulated so there is no suitable miRNA to select in this instance.*

*Two unchanged miRNAs were also validated. First, miR-16-5p which was used as the comparator for the assays. Second, miR-146a-5p which was below the 1.5-fold cut-off in the microarray; validation of this unchanged miRNA is now included as Figure 1D. Therefore I believe the technical validity of the microarray is not in question.*

No functional readouts are included for any of the experiments (for example, phagocytic activity or M1/M2 polarization) upon miR-155-3p OE or knockdown.

*Indeed, such experiments were not performed, so this data cannot be provided retrospectively. The work sought to examine the responses to the TLR4 signalling pathway rather than macrophage polarisation per se, that latter of which is achieved using combinations of stimuli/cytokines that were not used. TNF secretion was used as a positive control for TLR4 stimulation, and expected condition of the unstimulated cells (ie lacking in TNF secretion). This has been explained in the final paragraph of the introduction.*

Although it is claimed that miR-155 is one of the most well-known miRNA involved in immune responses, the data in the paper suggests that at least in MDM, it does not play an important role in TNF secretion. This point needs further clarification.

*TNF secretion is only one aspect of the immune response, as laid out in the introduction other immune functions of miR-155 are in the adaptive immune system. Others (eg Kurowska-Stolarska et al PNAS 2011) have shown that miR-155-5p can modulate TNF secretion, however these authors studied longer timepoints (24hrs) than here. A discrepancy is perhaps not surprising given that miR-155-5p is much more highly expressed at this later timepoint, however these kinetic differences are out of the scope of the current manuscript that focussed on the primary response at early timepoints. This point has been emphasised in a new paragraph in the discussion.*

Introduction - Paragraph 2 Should read "BIC knockout causes defects in germinal center formation and Ig class switching, leading to immunodeficiency".

*Corrected*

Methods, Inhibition of miRNAs; Last sentence needs rephrasing or clarification "since pilot experiments showed that extending the recovery time to significantly reduced the efficiency of inhibition below a meaningful level."

*Rephrased*

Results

Figure 2C) Unclear why the author uses a crude extract of the toxin, when results from a pure synthetic version are already presented.

*This finding may be of interest to the Buruli ulcer research community, where mycolactone is the pathogenic determinant of the extensive necrosis and immune suppression in that disease. Mycolactone purified from bacteria likely includes some natural variation in hydroxylation due to leakiness of the polyketide synthase machinery. I have therefore retained this data in the revised version.*

Figure 3C) This figure should read "Primary monocytes" instead of "Primary monocytes". 

*Typo corrected*

Discussion; Being a sole author, it is understandable that there are physical limitations to the number of assays that can be performed on primary cells. But this is an obvious condition, making it unnecessary to reiterate it in the discussion section. The connection between miR-155 and immune activation in the model system used still remains unclear and needs further literature to support a model.

*The discussion now includes a concluding paragraph that places the work in context. As mentioned above, since the results being reported in the unique format of Wellcome Open Research do not include functional analysis; therefore I have been careful not to overstate the findings. Proposing a speculative model that isn’t supported by the current data would fall into this category.*

# Peer review: Reviewer response for version 1

**DOI:** 10.21956/wellcomeopenres.16435.r35300

**Published:** 2019-04-24

**Authors:** Gracjan Michlewski

**Affiliations:** 1 Division of Infection and Pathway Medicine, University of Edinburgh, Edinburgh, UK; 2 Zhejiang University - University of Edinburgh Institute, Haining, China

RE Simmonds presents a study on the role of LPS on human monocyte-derived macrophages in the context of regulation of miRNA abundance. The author first analysed relative levels of miRNA during LPS stimulation by microarray technology. This resulted in identification of several miRNAs that were upregulated more than 1.5 fold upon LPS treatment. The most upregulated was miR-155-3p, derived from a pri-miR-155 (B cell integration cluster RNA - BIC). Absolute quantification of miR-155-3p. miR-155-5p and BIC showed intricate, time-dependent regulation of their abundance upon LPS stimulation. Intriguingly, other pathogen-associated molecular patterns also influenced miR-155-3p. miR-155-5p and BIC abundance but with various profiles. Finally, the author wanted to see if one of the predicted miR-155-3p target (TNF) was affected by the miR-155-5p or miR-155-3p inhibition. In general, it's an interesting study that shows fast and substantial response from the miRNAs to LPS stimulation. That said, few important controls are missing, the research into miRNA biogenesis is not adequately cited, some figure panels are not referenced in the text and most importantly there are other possible explanations for the observed phenotypes that have to be considered.

 My specific comments are as follow:

1. Please expand the CD14 and MD2 abbreviations.
2. It would be good to provide GO term analysis of miR-155-5p and miR-155-3p predicated and validated targets. This might help to see a broader picture of what these miRNAs regulate in the context of innate immune response.
3. "...miR-155-3p was >100-fold induced at 2 hours, after which the levels began to reduce again (Figure 1D)..." I am pretty sure this is a reference to Figure 1E.
4. "...following exposure of macrophages to LPS were monitored alongside miR-155-5p and miR-155-3p in 10 human donors, taking a more detailed look at either early or later timepoints following stimulation (compiled data, Figure 1E)..." This is a reference to Figure 1F.
5. The main conclusion of the paper is that the biogenesis of miR-155-5p and miR-155-3p is altered during LPS stimulation. Unfortunately, there is no evidence for that. One possible way to get this information would be to perform *in vitro* pri-miRNA and pre-miRNA processing assays (before and after LPS stimulation).
  Also, one could analyse the levels of pre-miR-155 upon LPS stimulation. This could add additional layer of information about which biogenesis step (if any) is regulated.
6. There is another possible explanation for the observed phenotypes. It is possible that while miR-155-3p is upregulated in line with the BIC levels, the stability of miR-155-5p is compromised after LPS stimulation. To assay that, one would have to inhibit pol II (by actinomycin D) and trace the levels of miR-155-5p and miR-155-3p to calculate their half-life with and without LPS stimulation.
7. I don't understand the use of cycloheximide? I would rather see inhibition of pol II by actinomycin D (as described before) to see if the regulation of miR-155 is uncoupled from the regulation of transcription.
8. The reference to Figure 3D is missing.
9. The references to papers in miRNA biogenesis filed are completely missing. There is a mention of the KHSRP protein regulating miRNA biogenesis but no reference provided. My advice is to at least cite some recent comprehensive reviews on this subject (Treiber *et al*. 2019 ^1^, Michlewski and Caceres 2019 ^2^ and Creugny *et al*. 2018 ^3^).
10. The author reports that there were failed attempts to validate predicted mRNA targets for miR-155-3p function. This is a wrong approach. The focus should be on the proteins that these mRNAs code as the effects of miR-155-3p could be mostly visible at the level of protein translation.

I confirm that I have read this submission and believe that I have an appropriate level of expertise to confirm that it is of an acceptable scientific standard, however I have significant reservations, as outlined above.

## References

[1] : Regulation of microRNA biogenesis and its crosstalk with other cellular pathways. *Nat Rev Mol Cell Biol*.2019;20(1) : 10.1038/s41580-018-0059-1 5-20 10.1038/s41580-018-0059-1 30228348

[2] : Post-transcriptional control of miRNA biogenesis. *RNA*.25(1) : 10.1261/rna.068692.118 1-16 10.1261/rna.068692.118 30333195PMC6298569

[3] : Regulation of primary microRNA processing. *FEBS Lett*.592(12) : 10.1002/1873-3468.13067 1980-1996 10.1002/1873-3468.13067 29683487

# Response

**Published:** 2019-09-06

**Authors:** Rachel Simmonds

**Affiliations:** University of Surrey, UK

*Thank you for taking the time to review my manuscript. As mentioned in the introduction, it reports historical data (several years past). While I agree that the additional experiments suggested would give greater insight to the data, there is no prospect of performing them as the lab where the work was done no longer exists (please see the acknowledgements). Hence this submission to WOR, whose unique approach of seeking to publish “results worth sharing” rather than complete stories has given a route to release the data and methodology within, that otherwise would have remained unpublished.*

*I have therefore modified the text to reflect your concerns and the additional/alternative approaches suggested, and have looked closely at the manuscript overall to ensure that the conclusions are not overstated on the basis of the available data.*

My specific comments are as follow:

Please expand the CD14 and MD2 abbreviations.

*Expanded as requested*

It would be good to provide GO term analysis of miR-155-5p and miR-155-3p predicated and validated targets. This might help to see a broader picture of what these miRNAs regulate in the context of innate immune response.

*I have performed GO term analysis from miRDB predicted targets for miR-155-5p and miR-155-3p using the Panther over-representation test and the annotation dataset of Panther pathways. This is now presented as Figure 1H, and next text describes the pathways relevant to innate immunity. I’m not convinced that it’s terribly helpful. The recent and extensive review in original reference 6, Alivernini et al has focussed on the currently known validated targets, and I have included a more detailed description of the broader picture and cited this reference again at the end of the discussion.*

"...miR-155-3p was >100-fold induced at 2 hours, after which the levels began to reduce again (Figure 1D)..." I am pretty sure this is a reference to Figure 1E.

"...following exposure of macrophages to LPS were monitored alongside miR-155-5p and miR-155-3p in 10 human donors, taking a more detailed look at either early or later timepoints following stimulation (compiled data, Figure 1E)..." This is a reference to Figure 1F.

*My apologies for the mislabelling. This has been corrected, taking into account the addition of further validation data requested by another reviewer.*

The main conclusion of the paper is that the biogenesis of miR-155-5p and miR-155-3p is altered during LPS stimulation. Unfortunately, there is no evidence for that. One possible way to get this information would be to perform in vitro pri-miRNA and pre-miRNA processing assays (before and after LPS stimulation).

*I was very careful to avoid making this conclusion, as I agree that further work would be needed to support such a contention. It is not the conclusion that was stated in the original abstract nor the discussion. However, in response to your comment I have reworded these to ensure that this cannot be misunderstood by other readers.*

Also, one could analyse the levels of pre-miR-155 upon LPS stimulation. This could add additional layer of information about which biogenesis step (if any) is regulated.

*I agree that analysing the abundance of the pre-miR-155 hairpin would be ideal, and I have looked again to see if this would be possible and concluded the following:*

- *No commercial suppliers make a validated assay for pre-miR-155*
- *Previous papers (eg O’Connell PNAS 2007) find that pre-miR-155 is barely detectable at any timepoint before or during LPS stimulation of BMDM by Northern blot*
- *Very few papers report quantifying pre-miR-155, and in call cases they use in-house SYBR-based PCR that cannot technically discriminate between pre-miR-155 and pri-miR-155 (PCR of the stemloop sequence would amplify both the pre-miR and pri-miR) *
- *In those papers that do directly compare pri-miR and pre-miR abundance for miR-155, they show that the assays give broadly comparable results (not surprising given the above)*
- *Other studies of miR-155-5p and -3p biogenesis (eg Zhou et al, Blood 2010, Ruggiero et al FEBS Lett 2019) also use pri-miR-155 assays to quantify the precursors, as I did.*

*Given that it is not possible to generate further samples for alternate approaches such as Northern blotting, I have made modifications to the results and discussion in light of the above. Furthermore, taking the approach of Zhou *et al *I have relabelled “BIC” as “pri-miR-155” in all figures and text, to clearly indicate the precursors that have been quantified.*

There is another possible explanation for the observed phenotypes. It is possible that while miR-155-3p is upregulated in line with the BIC levels, the stability of miR-155-5p is compromised after LPS stimulation. To assay that, one would have to inhibit pol II (by actinomycin D) and trace the levels of miR-155-5p and miR-155-3p to calculate their half-life with and without LPS stimulation.

*This would indeed be an interesting experiment, complicated by the fact that miR-155-5p itself is able to stabilise mRNA transcripts such as TNF (see Bala et al JBC 2011 p1436) and that miR-155-5p was recently shown to have a half-life of 10.5hrs (Marzi et al, Genome Res 2016 p554). Actinomycin D would surely have killed cells after this period of exposure, where such experiments usually run for a  couple of hours a most. Such notions of miRNA degradation and stability are only recently emerging and tackling this issue is out of the scope of the current manuscript. However, a discussion of this alternative explanation of the data is now given in the results and discussion.*

I don't understand the use of cycloheximide? I would rather see inhibition of pol II by actinomycin D (as described before) to see if the regulation of miR-155 is uncoupled from the regulation of transcription.

*As already mentioned in the text, cycloheximide is a standard test to separate out responses that required protein synthesis (secondary response) from that that done (primary response). I have now clarified this in the introduction.*

The reference to Figure 3D is missing.

*Corrected*

The references to papers in miRNA biogenesis filed are completely missing. There is a mention of the KHSRP protein regulating miRNA biogenesis but no reference provided. My advice is to at least cite some recent comprehensive reviews on this subject (Treiber et al. 2019, Michlewski and Caceres 2019 and Creugny et al. 2018).

*With respect, one of the references you provide (Creugney et al) was already citied (reference 1). I have included your review and the other reference as requested.*

The author reports that there were failed attempts to validate predicted mRNA targets for miR-155-3p function. This is a wrong approach. The focus should be on the proteins that these mRNAs code as the effects of miR-155-3p could be mostly visible at the level of protein translation.

*Indeed, a variety of approaches are possible to achieve this, and several were used. The advantage of using mRNA rather than protein abundance is that it is possible to screen a larger number of potential candidates. Since miRNAs regulated both by inhibiting translation and by causing target mRNAs to be degraded this approach is acceptable. Never-the-less I have re-edited the discussion section to highlight different areas, and expand on the study limitations in response to all reviewer comments and the manuscript no longer refers to these failed attempts.*
